# A Lytic Polysaccharide Monooxygenase with Broad Xyloglucan Specificity from the Brown-Rot Fungus Gloeophyllum trabeum and Its Action on Cellulose-Xyloglucan Complexes

**DOI:** 10.1128/AEM.01768-16

**Published:** 2016-10-27

**Authors:** Yuka Kojima, Anikó Várnai, Takuya Ishida, Naoki Sunagawa, Dejan M. Petrovic, Kiyohiko Igarashi, Jody Jellison, Barry Goodell, Gry Alfredsen, Bjørge Westereng, Vincent G. H. Eijsink, Makoto Yoshida

**Affiliations:** a Department of Environmental and Natural Resource Science, Tokyo University of Agriculture and Technology, Fuchu, Tokyo, Japan; b Department of Chemistry, Biotechnology and Food Science, Norwegian University of Life Sciences (NMBU), Ås, Norway; c Department of Biomaterial Sciences, Graduate School of Agriculture and Life Sciences, The University of Tokyo, Tokyo, Japan; d VTT Technical Research Centre of Finland, Espoo, Finland; e Center for Agriculture, Food and the Environment, University of Massachusetts, Amherst, Massachusetts, USA; f Department of Sustainable Biomaterials, Virginia Polytechnic Institute and State University, Blacksburg, Virginia, USA; g Norwegian Institute of Bioeconomy Research, Ås, Norway; HKI and University of Jena

## Abstract

Fungi secrete a set of glycoside hydrolases and lytic polysaccharide monooxygenases (LPMOs) to degrade plant polysaccharides. Brown-rot fungi, such as Gloeophyllum trabeum, tend to have few LPMOs, and information on these enzymes is scarce. The genome of G. trabeum encodes four auxiliary activity 9 (AA9) LPMOs (*Gt*LPMO9s), whose coding sequences were amplified from cDNA. Due to alternative splicing, two variants of *Gt*LPMO9A seem to be produced, a single-domain variant, *Gt*LPMO9A-1, and a longer variant, *Gt*LPMO9A-2, which contains a C-terminal domain comprising approximately 55 residues without a predicted function. We have overexpressed the phylogenetically distinct *Gt*LPMO9A-2 in Pichia pastoris and investigated its properties. Standard analyses using high-performance anion-exchange chromatography–pulsed amperometric detection (HPAEC-PAD) and mass spectrometry (MS) showed that *Gt*LPMO9A-2 is active on cellulose, carboxymethyl cellulose, and xyloglucan. Importantly, compared to other known xyloglucan-active LPMOs, *Gt*LPMO9A-2 has broad specificity, cleaving at any position along the β-glucan backbone of xyloglucan, regardless of substitutions. Using dynamic viscosity measurements to compare the hemicellulolytic action of *Gt*LPMO9A-2 to that of a well-characterized hemicellulolytic LPMO, *Nc*LPMO9C from Neurospora crassa revealed that *Gt*LPMO9A-2 is more efficient in depolymerizing xyloglucan. These measurements also revealed minor activity on glucomannan that could not be detected by the analysis of soluble products by HPAEC-PAD and MS and that was lower than the activity of *Nc*LPMO9C. Experiments with copolymeric substrates showed an inhibitory effect of hemicellulose coating on cellulolytic LPMO activity and did not reveal additional activities of *Gt*LPMO9A-2. These results provide insight into the LPMO potential of G. trabeum and provide a novel sensitive method, a measurement of dynamic viscosity, for monitoring LPMO activity.

**IMPORTANCE** Currently, there are only a few methods available to analyze end products of lytic polysaccharide monooxygenase (LPMO) activity, the most common ones being liquid chromatography and mass spectrometry. Here, we present an alternative and sensitive method based on measurement of dynamic viscosity for real-time continuous monitoring of LPMO activity in the presence of water-soluble hemicelluloses, such as xyloglucan. We have used both these novel and existing analytical methods to characterize a xyloglucan-active LPMO from a brown-rot fungus. This enzyme, *Gt*LPMO9A-2, differs from previously characterized LPMOs in having broad substrate specificity, enabling almost random cleavage of the xyloglucan backbone. *Gt*LPMO9A-2 acts preferentially on free xyloglucan, suggesting a preference for xyloglucan chains that tether cellulose fibers together. The xyloglucan-degrading potential of *Gt*LPMO9A-2 suggests a role in decreasing wood strength at the initial stage of brown rot through degradation of the primary cell wall.

## INTRODUCTION

For decades, the enzymatic degradation of cellulose by filamentous fungi was considered to proceed through the hydrolytic action of cellulases. As early as in 1950, the involvement of additional factors in cellulose conversion was proposed (1), and in 1974, Eriksson et al. showed that enzymatic hydrolysis of cellulose by a crude fungal enzyme mixture is promoted by the presence of molecular oxygen, suggesting a role for redox reactions (2). The explanation for the observations by Eriksson et al. (2) came almost 4 decades later, when copper-dependent lytic polysaccharide monooxygenases (LPMOs), which are currently classified into auxiliary activity (AA) families 9, 10, 11, and 13 in the CAZy database (3, 4), were first described (5–7). Since their discovery, LPMOs received much attention and are currently considered one of the key enzymes in fungal cellulose degradation (8). LPMOs employ molecular oxygen and an external electron donor (5, 9–12) to carry out oxidative cleavage of the β-1,4-glucosidic bonds in cellulose. Some LPMOs exclusively oxidize C-1, others exclusively oxidize C-4, whereas a third type of LPMOs yields a mixture of C-1- and C-4-oxidized products. Recently, LPMOs have been found to show oxidative activity against various other plant polysaccharides, including xyloglucan, glucomannan, xylan, and starch (13–19). Thus, the physiological roles of LPMOs in biomass decomposition are likely to be more complex and varied than initially assumed.

Brown-rot fungi are a group of wood-rotting basidiomycetous fungi and represent the dominant wood decay fungi in northern coniferous forest ecosystems. They are able to remove plant cell wall polysaccharides, such as cellulose and hemicelluloses, together with extensive lignin depolymerization and modification, but without lignin metabolism (20–22). Brown-rot fungi employ a unique system of wood degradation, in which they combine enzymatic and chemical mechanisms. The chemical mechanism implies the formation of hydroxyl radicals (·OH) through a chelator-mediated Fenton (CMF) reaction that randomly attacks the wood cell wall components (22–26). This process causes structural and chemical changes in the wood cell wall, potentially providing carbohydrate-active enzymes with greater access to the substrate by generating holes large enough for the enzymes to infiltrate the cell wall (21). Compared to soft-rot and white-rot fungi, the majority of brown-rot fungi seem to have an incomplete enzymatic system for cellulose degradation. In particular, they do not produce processive cellobiohydrolases, which are key enzymes in the degradation of crystalline cellulose (27–29). Brown-rot fungi also often lack cellobiose dehydrogenase (CDH), which is considered to be a natural electron donor of fungal LPMOs (9, 10, 12), albeit not the only one (11, 30, 31). While several cellulases and hemicellulases from brown-rot fungi have been characterized, little is known about brown-rot LPMOs (32). In general, the physiological function of oxidative enzyme systems in wood degradation by brown-rot fungi is still unclear.

Currently, the detection of LPMO activity on polymeric substrates is based on measurement of the production of soluble oligomeric products, which are released as a result of LPMO cleavage near polymer chain ends or cleavage of the same polymer chain twice at nearby positions. Such products can be detected using various types of chromatography (33, 34) or mass spectrometry (5). The activity of strictly C-4-oxidizing LPMOs may also be quantified directly using a standard method for detection of the newly generated reducing ends (13). Recently, a semiquantitative high-throughput method for screening activity toward water-soluble substrates has been reported (35), which is suitable for a wide variety of carbohydrate-acting enzymes and can be adapted for LPMOs (13). LPMO action on solid substrates may also be quantified using confocal laser scanning microscopy after labeling C-1-oxidized cellulose chain ends with a fluorescence dye that is specific for carboxylic acids (36). Unlike for endo-acting glycoside hydrolases, the use of gel permeation chromatography or viscosity measurements for detecting LPMO-generated decreases in the molecular weight of carbohydrate polymers has not yet been reported. Such analytical tools potentially reveal activities that cannot be detected by the other available methods and are also of industrial relevance because they relate to (reducing) biomass viscosity.

In the present study, we cloned five cDNAs encoding putative LPMO9s from Gloeophyllum trabeum (*Gt*LPMO9s), which is one of the most studied brown-rot fungi. We heterologously expressed the one LPMO that, by phylogenetic analysis, seemed distant from most other known LPMOs, namely, *Gt*LPMO9A-2. This enzyme has the longer amino acid sequence of the two naturally occurring variants of *Gt*LPMO9A (see below) and was expressed in Pichia pastoris. As part of the in-depth characterization of *Gt*LPMO9A-2, we developed a method for monitoring LPMO activity on hemicelluloses by measuring the reduction in viscosity. We show that *Gt*LPMO9A-2 is a promiscuous LPMO with a unique ability to cleave xyloglucan regardless of the substitutions of the β-1,4-linked glucan backbone. Using dynamic viscosity measurements, we were able to detect activity on additional hemicelluloses that remained undetected in the standard chromatographic and mass spectrometry analyses.

## MATERIALS AND METHODS

### Strains and enzymes.

Gloeophyllum trabeum strain NBRC 6430 was used as a source of LPMO genes. Escherichia coli strain JM109 (TaKaRa Bio, Shiga, Japan) and Pichia pastoris strain KM71H (Invitrogen, Carlsbad, CA) were used as hosts for subcloning experiment and heterologous production of recombinant *Gt*LPMO9A-2, respectively.

The recombinant LPMO9C from Neurospora crassa (*Nc*LPMO9C; UniProt accession no. Q7SHI8) was prepared according to Kittl et al. (37). Endoglucanases Cel12A from Aspergillus fumigatus (*Af*Cel12A; UniProt accession no. Q4WGT4) and Cel5A from Thermoascus aurantiacus (*Ta*Cel5A; UniProt accession no. Q8TG26) were produced and purified as previously described (38).

### Sequence analysis.

Multiple-sequence alignments were generated using MAFFT, version 7.295 (39), available at the European Bioinformatics Institute website. For phylogenetic analysis of LPMO9s, amino acid sequences were aligned using MAFFT and then manually edited using Sea View (40). A phylogenetic tree was generated from this alignment by the neighbor-joining method (41) in the ClustalX software (42), with 1,000 bootstraps, as previously described (43).

### Cloning of genes encoding *Gt*LPMO9s.

G. trabeum was cultivated in Highley's medium (44) containing 0.5% glucose (Wako, Osaka, Japan) as the sole carbon source, and without thiamine hydrochloride. After 7 days of cultivation at 23°C and 120 rpm, total RNA was extracted using the RNeasy plant minikit (Qiagen, Venlo, The Netherlands). First-strand cDNA was then synthesized using reverse transcriptase (SuperScript III; Invitrogen) with 3′ rapid amplification of the cDNA ends (3′-RACE) using the GeneRacer kit (Invitrogen), according to the manufacturer's instructions; the 3′-RACE primers used are listed in Table 1. The regions encoding the mature LPMOs were sequenced after PCR amplification from first-strand cDNA using the open reading frame (ORF) primers. For expression, the insertion fragments of the *Gt*LPMO9s, except for *Gt*LPMO9A-2, were amplified by expression primers appending the XhoI site and the Kex2 cleavage site at the 5′ end and the NotI site at the 3′ end of the coding sequences without signal peptide (Table 1) and cloned into pPICZα (Invitrogen) by restriction digestion and ligation. After amplification with expression primers, the insertion fragment of *Gt*LPMO9A-2 was inserted into pPICZα (Invitrogen) using the In-Fusion HD cloning kit (TaKaRa Bio); this strategy was chosen because the ORF encoding *Gt*LPMO9A-2 contains a NotI cleavage site.

**TABLE 1:**
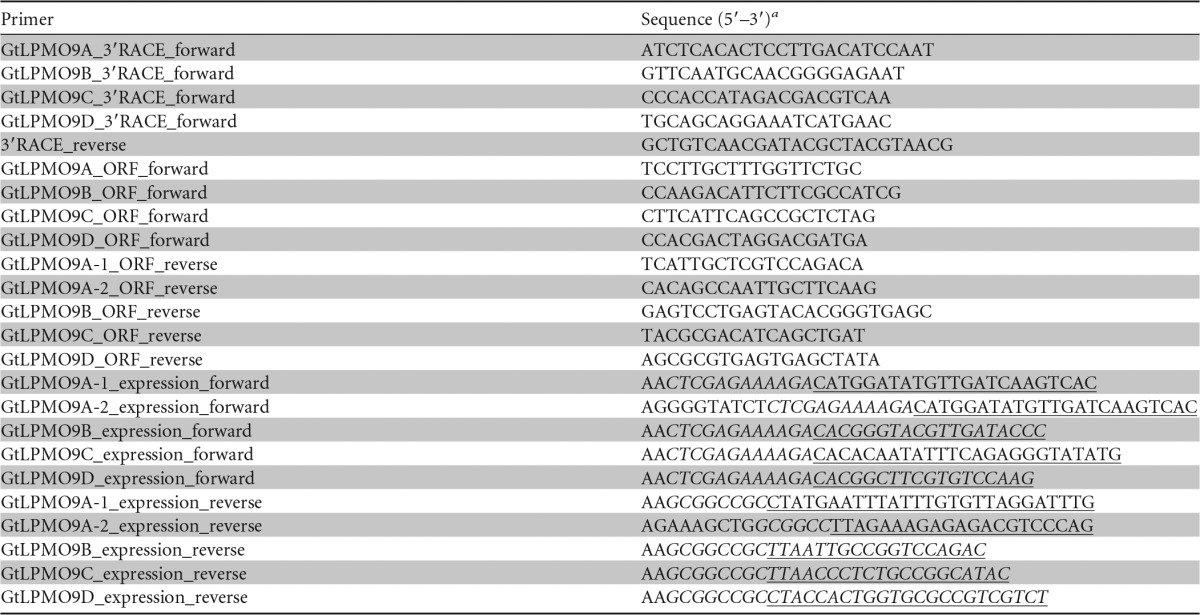
Oligonucleotide primers

^a^ The restriction sites XhoI (*CTCGAG*, for forward expression primers) and NotI (*GCGGCCGC*, for reverse expression primers [only partial in the *Gt*LPMO9A-2 primer]) and the sequence encoding the cleavage site of Kex2 (*AAAAGA*, encoding Lys-Arg in forward primers) are printed in italics. ORF sequences are underlined. Expression primers were designed with a protecting AA sequence at the 5′ end. *Gt*LPMO9A-2 expression primers, for In-Fusion cloning, were designed to contain 15 extra bases at the 5′ end, overlapping the Kex2 (forward) or NotI restriction cleavage site (reverse) of pPICZα.

### Heterologous expression of the *Gt*LPMO9s and purification of *Gt*LPMO9A-2.

Approximately 10 μg of pPICZα expression plasmid DNA was linearized with *Bpu*1102I (TaKaRa Bio) prior to transformation into P. pastoris. Electroporation, selection of transformants, and production of recombinant protein were carried out according to the instruction manual of the EasySelect Pichia expression kit (Invitrogen). After cultivating for 4 days in YP medium using methanol as the carbon source, the cells were removed by centrifugation (30 min at 10,000 × *g*), and then the supernatants were applied for SDS-PAGE analysis to evaluate the production of recombinant proteins.

For purification of *Gt*LPMO9A-2, all steps were carried out at 4°C, unless indicated otherwise. The P. pastoris strain expressing *Gt*LPMO9A-2 was grown in 4 liters of YP medium containing 1% glycerol for 1 day at 30°C and 180 rpm. The cells were collected by centrifugation at 1,500 × *g* for 5 min, resuspended in 400 ml of YP medium containing 1% (vol/vol) methanol to induce expression, and incubated further at 30°C. Every 24 h, methanol was added to the culture to a final concentration of 1% (vol/vol). After 4 days, the cell-free supernatant was harvested by centrifugation at 10,000 × *g* for 30 min. Saturated ammonium sulfate solution was added to the cell-free broth to a final concentration of 50% (wt/wt). After removing the precipitate by centrifugation (30 min at 10,000 × *g*), the supernatant was diluted approximately 2.2 times with 20 mM sodium acetate buffer (pH 4.5) to a final concentration of approximately 1 M ammonium sulfate, and applied to a Toyopearl Phenyl-650S column (20 mm [diameter] by 160 mm; Tosoh, Tokyo, Japan) equilibrated with 20 mM sodium acetate buffer (pH 4.5) containing 1 M ammonium sulfate. The protein was eluted by applying a 100-ml linear reverse gradient to 20 mM sodium acetate buffer (pH 4.5). Fractions containing the recombinant protein were collected and pooled, and the buffer was changed to 20 mM sodium acetate buffer (pH 4.0) using Vivaspin 20 centrifugal concentrator tubes with a 10,000-molecular-weight cutoff (MWCO) membrane (Sartorius AG, Göttingen, Germany). Then, the sample was applied to a Toyopearl SP-650S column (20 mm [diameter] by 250 mm; Tosoh) equilibrated with the same buffer. The flowthrough containing the recombinant protein was collected, the buffer was changed to 20 mM sodium acetate buffer (pH 5.5) with Vivaspin 20 centrifugal concentrator tubes, and ca. 13.1 mg of protein was treated with 1 μl of endo-β-*N*-acetylglucosaminidase H (Endo H) (500 U; New England BioLabs, Ipswich, MA) at 30°C overnight. The protein solution buffer was then exchanged with 20 mM Tris-HCl buffer (pH 7.0) with Vivaspin 20 centrifugal concentrator tubes and applied to a Toyopearl DEAE-650S column (20 mm [diameter] by 250 mm; Tosoh) equilibrated with the same buffer. The recombinant protein was eluted from the column with a linear NaCl gradient, and the fractions eluting from 28 to 52 mM NaCl were collected. Protein purity was analyzed by SDS-PAGE analysis with 12% polyacrylamide gels, and the N-terminal amino acid sequence was determined with a protein sequencer (model 491 cLC; Applied Biosystems, Foster City, CA).

### Substrates.

The following substrates were used to characterize the substrate specificity of *Gt*LPMO9A-2: phosphoric acid swollen cellulose (PASC), carboxymethyl cellulose (CMC), cellohexaose, cellopentaose, tamarind xyloglucan (partially arabinosylated [XG]), xyloglucan oligosaccharide (XG oligomers), and xyloglucan heptasaccharide (XG7), konjac glucomannan (GM), ivory nut mannan, wheat arabinoxylan, oat flour mixed-linkage (β-1,3–1,4) glucan, oat spelt xylan, beech wood xylan, and birchwood xylan. PASC was prepared as described earlier (45); birchwood xylan was purchased from Carl Roth GmbH (Karlsruhe, Germany). Konjac glucomannan used in the dynamic viscosity experiments was purchased from Wako; konjac glucomannan used in the coating experiments was purchased from Megazyme (Wicklow, Ireland). Oat spelt xylan was purchased from Serva Electrophoresis GmbH (Heidelberg, Germany), and beech wood xylan was purchased from Sigma-Aldrich (St. Louis, MO). All other substrates were obtained from Megazyme.

### LPMO activity measurements: dynamic viscosity experiments.

Dynamic viscosity of the reaction solution was determined using an AMVn automated microviscometer (Anton Paar, Graz, Austria), which is a falling ball-type viscometer measuring the rolling time of a ball through a liquid in a capillary. Reaction mixtures of 1 ml in total contained 1 μM copper-saturated enzyme (see below), 0.5 mM dithiothreitol (DTT), and substrate in 50 mM sodium acetate buffer (pH 5.0). The concentration of each substrate was adjusted, based on its intrinsic viscosity, so that the viscosity falls within the optimal range of the viscometer. The concentrations of konjac glucomannan, tamarind xyloglucan, arabinoxylan, and CMC were 0.05%, 0.15%, 0.2%, and 0.5% (wt/vol), respectively. The reactions were carried out inside a glass capillary (1.6 mm [diameter]) containing a steel ball (1.5 mm [diameter]) thermostated at 30°C. The capillary was positioned at a 50° angle relative to horizontal (causing the ball to move through the reaction mixture along the capillary) and was automatically inverted when the ball reached its lower end. The rolling time of the steel ball was continuously recorded with the viscometer while the capillary was inverted 2,000 times. Endpoint samples (after 16 h of incubation, when the change in viscosity had leveled off) were analyzed by high-performance anion-exchange chromatography–pulsed amperometric detection (HPAEC-PAD) and matrix-assisted laser desorption ionization–time of flight mass spectrometry (MALDI-TOF MS) to check for the formation of soluble oxidized oligosaccharides, as described below.

Dynamic viscosity was calculated as *K* × (ρ − ρ_0_) × *T*, where *K* is the calibration constant of the system (in millipascals-centimeters cubed per gram), ρ is the density of the ball (in grams per centimeter cubed), ρ_0_ is the density of the reaction mixture (in grams per centimeter cubed), and *T* is the rolling time of the ball (in seconds). The calibration constant *K* was experimentally determined to be 0.00845 mPa · cm^3^/g, using water as a standard.

The dynamic viscosity data were fit to an exponential decay formula (*y* = *a* × e^[^^*b* × *x*]^ + *c*) with DeltaGraph (SPSS, Inc., CA), where *y* is the dynamic viscosity, *x* is the time, and *a*, *b*, and *c* are constants. The constant *c* is the final viscosity of the substrate. The initial decline rate of dynamic viscosity was calculated by differentiating the formula with respect to *x* = 0. In the case of arabinoxylan and CMC, the correlation between dynamic viscosity and time was linear.

### Endoglucanase treatment.

To convert oxidized products into short oligosaccharides, the endpoint samples of the dynamic viscosity experiments were subjected to endoglucanase treatment, and the resulting oligomer mixtures were analyzed by HPAEC-PAD and MALDI-TOF MS (see below). Purified endoglucanases, *Af*Cel12A and *Ta*Cel5A, were diluted to a concentration of 100 μM, and 1 μl of *Af*Cel12A or *Ta*Cel5A solution was added to 50 μl of the reaction mixtures containing LPMO-treated xyloglucan or glucomannan. Subsequently, the reaction mixtures were incubated for 20 h at 30°C.

### Screening for substrate specificity.

After Cu(II) saturation (46), *Gt*LPMO9A-2 (or *Nc*LPMO9C) was incubated with various substrates in a 100-μl total reaction volume containing 1 μM enzyme, 0.5 mM dithiothreitol (DTT), or 1 mM ascorbic acid (ASC), as well as the substrate in 50 mM sodium acetate buffer (pH 5.0). The concentrations of substrates were as follows: 0.2% (wt/vol) PASC or Avicel, 0.2% (wt/vol) CMC, 0.05% (wt/vol) GM, 0.15% (wt/vol) XG, 0.2 g/liter cellopentaose, cellohexaose, XG7, or XG oligomer, and 0.5% (wt/vol) mannan, xylan, or mixed-linkage glucan. The reaction mixtures were incubated at 30°C in an Eppendorf ThermoMixer C (Eppendorf AG, Hamburg, Germany) with shaking at 1,000 rpm for 16 h. The reactions were run at least in duplicate, the reaction mixtures were boiled for 10 min after sampling, and the solids were separated by centrifugation at 10,000 × *g* for 5 min. The supernatants were analyzed by HPAEC-PAD and MALDI-TOF MS (see below) to check for the formation of soluble oxidized oligosaccharides.

### Activity on complex substrates.

In order to evaluate LPMO activity on complex substrates, cellulose was coated with hemicellulose by premixing an aqueous solution of 1% (wt/vol) tamarind XG or 1% (wt/vol) konjac GM with 1% (wt/vol) PASC in a 1:1 (vol/vol) ratio for 15 min (at room temperature without shaking, in 20 mM Na-acetate buffer [pH 5.0]) prior to enzyme addition. After the preincubation reactions, reaction mixtures were prepared in 20 mM Na-acetate buffer (pH 5.0) containing 0.2% (wt/vol) PASC and 0.2% (wt/vol) XG or GM. Reaction mixtures containing 0.2% (wt/vol) of XG, GM, or PASC as a single substrate were also prepared. Further, the reaction mixtures contained 1 μM *Gt*LPMO9A-2 or *Nc*LPMO9C (copper saturated, according to Loose et al. [46]) and 1 mM ascorbic acid, and they were incubated at 30°C for 24 h. The reactions were run at least in duplicate, the reaction mixtures were boiled for 10 min after sampling, and the solids were separated by centrifugation at 10,000 × *g* for 5 min. Products were analyzed using HPAEC-PAD, as described below. Control experiments were performed without electron donor or enzyme.

### Detection of oxidized oligosaccharides.

The oligosaccharides released by LPMO action and sequential LPMO-endoglucanase treatment were analyzed by high-performance anion-exchange chromatography (HPAEC) on a Dionex ICS3000 system equipped with pulsed amperometric detection (PAD), using a 50-min gradient (33) for cellulosic substrates and a 75-min gradient (13) for hemicellulosic substrates.

The oligosaccharides were further analyzed using MALDI-TOF MS. The analysis was carried out on an Ultraflex MALDI-TOF/TOF instrument (Bruker Daltonics, Bremen, Germany) equipped with a nitrogen 337-nm laser beam, as described earlier (5). Samples (2 μl) were applied to an MTP 384 ground steel target plate TF (Bruker Daltonics) together with 4.5 mg of 2,5-dihydroxybenzoic acid (DHB) matrix dissolved in 0.5 ml of 30% acetonitrile. Data were collected with the lowest laser energy necessary to obtain sufficient quality spectra, using Bruker's flexControl software. Spectra were analyzed using Bruker's flexAnalysis software. All samples analyzed in this study contained 50 mM sodium acetate, which suppressed the formation of potassium adducts. The signal of amperometric detection is expressed in nanocoulombs (nC).

### Accession number(s).

The nucleotide sequences of the genes (cDNA) encoding *Gt*LPMO9A-1, *Gt*LPMO9A-2, *Gt*LPMO9C, and *Gt*LPMO9D have been deposited in the DDBJ database under accession numbers LC157847, LC157848, LC157849, and LC157850, respectively. The cDNA sequence of *Gt*LPMO9B has already been described by Jung et al. (32) and has been deposited in the GenBank database under accession no. AEJ35168 as an endo-β-1,4-glucanase (named Cel61G in the CAZy database).

## RESULTS

### Nucleotide and amino acid sequences.

The publicly available genome database of G. trabeum shows four genes encoding AA9 enzymes (29), which we have named *lmpo9A* (location in the G. trabeum genome database version 1.0: scaffold_00011:177323–178510), *lmpo9B* (scaffold_00011:179858–181138), *lmpo9C* (scaffold_00002:3232078–3233061), and *lmpo9D* (scaffold_00001:4158823–4160504). So far, only one of these genes, *lmpo9B*, has been cloned (called Cel61G in the CAZy database); studies of the recombinant protein have suggested that it acts on cellulose, but the enzyme remains largely uncharacterized (32). In the present study, the cDNA species transcribed from the *lpmo* genes were cloned by reverse transcription-PCR (RT-PCR) using total RNA extracted from mycelia of G. trabeum. The *lmpo9A* gene was transcribed into two different mRNAs (named *lmpo9A-1* and *lmpo9A-2*), the first 706 nucleotides of which were identical. The first 706 nucleotides were followed by another 53 nucleotides, corresponding to an exon for the shorter splicing variant (*Gt*LPMO9A-1) or an intron for the longer splicing variant (*Gt*LPMO9A-2). The longer transcript (*Gt*LPMO9A-2) continued with another 407 nucleotides (see Fig. S1 in the supplemental material).

The coding transcripts of *lmpo9A-1*, *lmpo9A-2*, *lmpo9B*, *lmpo9C*, and *lmpo9D* consist of 756, 1,110, 756, 768, and 1,032 bp, respectively, excluding the stop codon. Excluding the N-terminal signal peptide from the translated amino acid sequences, as predicted by the SignalP program (47), the mature *Gt*LPMO9A-2 protein consists of 351 amino acids, with a molecular mass of 36.0 kDa and a predicted pI of 4.56. For the other LPMOs, these values are: *Gt*LPMO9A-1, 233 amino acids, molecular mass of 25.1 kDa, and pI 4.94; *Gt*LPMO9B, 232 amino acids, molecular mass 24.5 kDa, and pI 6.19; *Gt*LPMO9C, 239 amino acids, molecular mass 25.8 kDa, and pI 4.68; and *Gt*LPMO9D, 321 amino acids, molecular mass 33.1 kDa, and pI 5.84.

**FIG 1:**
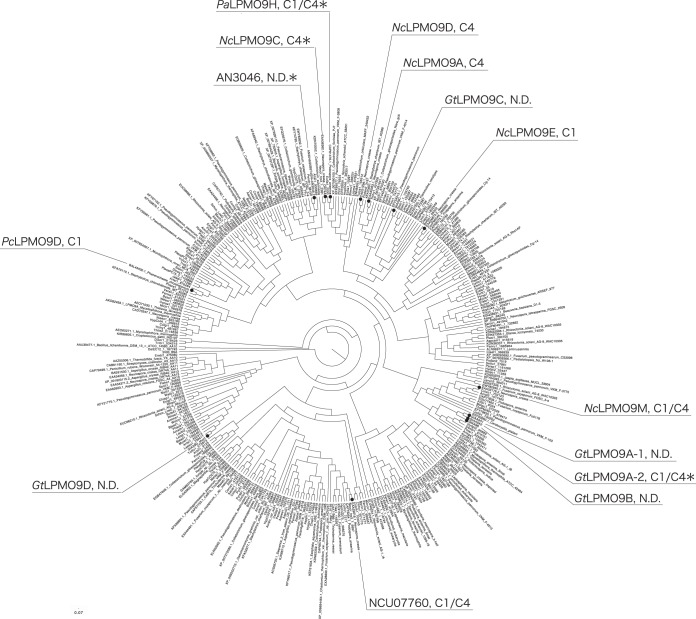
Position of G. trabeum LPMOs in the phylogenetic tree of LPMO9s. The phylogenetic tree was created from catalytic domains of LPMO9s by using the neighbor-joining method in ClustalX (version 2.1). Several LPMOs are labeled, and their cleavage specificities are indicated (C-1, C-4, or both [C-1/C-4]; N. D., not determined). For all labeled non-*Gt*LPMOs, activity on cellulose has been demonstrated. An asterisk indicates that additional activity on xyloglucan has been detected. The functional data for *Gt*LPMO9A-2 stem from the present report; other functional data come from references 13, 14, 16, 49, and 65).

**FIG 2:**
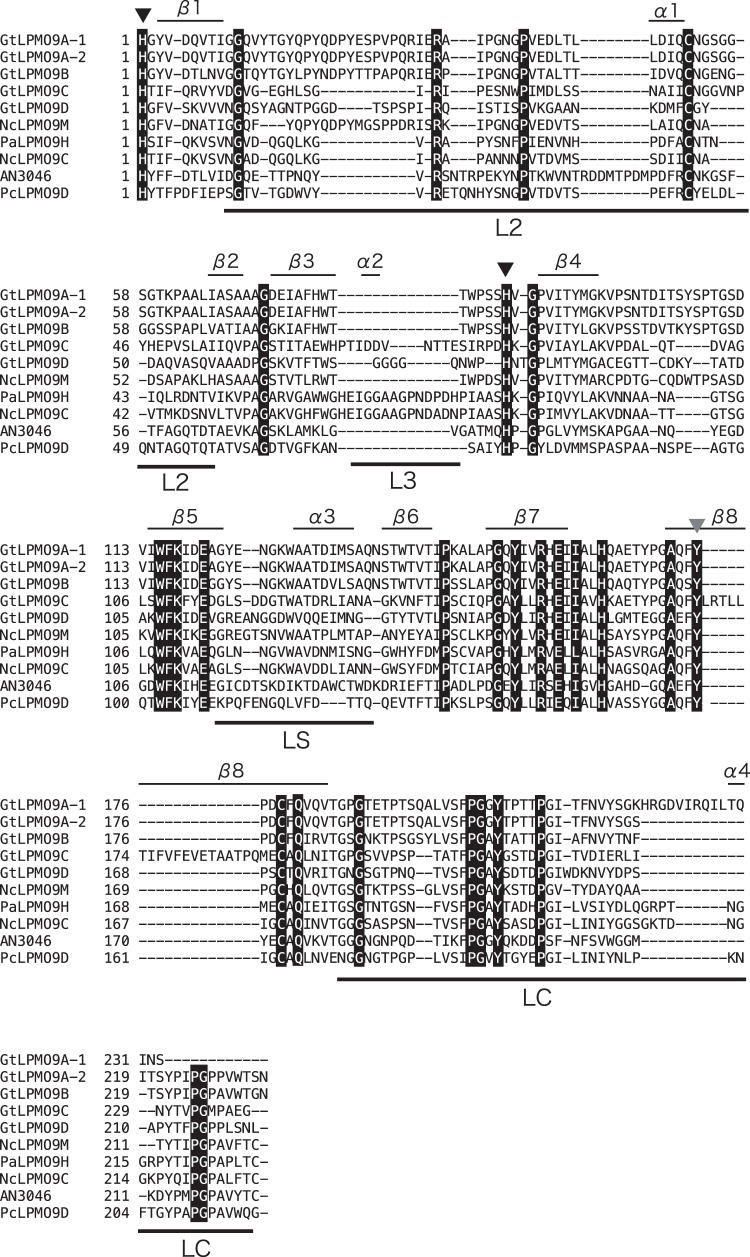
Multiple-sequence alignment of the catalytic domains of selected LPMO9s. Fully conserved residues are printed in white on a black background. Active-site histidines (black-filled triangles) and a tyrosine (gray-filled triangle) involved in copper coordination are indicated. The labeled bars over the sequences indicate known variable regions in LPMO9s (15, 51). Note that *Gt*LPMO9A-2 and *Gt*LPMO9D have C-terminal extensions; for more information, see Fig. S2 to S4 in the supplemental material. This alignment was generated using MAFFT, version 7.295 (39), available at the European Bioinformatics Institute website (https://www.ebi.ac.uk/Tools/msa/mafft/).

In a phylogenetic tree of LPMO9s, *Gt*LPMO9A-1, *Gt*LPMO9A-2, and *Gt*LPMO9B cluster in the same group, whereas *Gt*LPMO9C and *Gt*LPMO9D ended up in a different cluster each (Fig. 1). *Gt*LPMO9A-1, *Gt*LPMO9A-2, and *Gt*LPMO9B are closely related to a C-1/C-4-oxidizing LPMO from Neurospora crassa (*Nc*LPMO9M or NCU-07898 [48, 49]). Among characterized LPMOs, the ones being closest to *Gt*LPMO9C are the strictly C-4-oxidizing LPMOs *Nc*LPMO9A (NCU-02240 [49]), *Nc*LPMO9C (a well-characterized LPMO from Neurospora crassa with broad β-1,4-glucan-degrading abilities; NCU-02916 [13, 15, 50]), and *Nc*LPMO9D (NCU-01050 [48, 49]). *Gt*LPMO9D is part of a clade that seems quite distinct from all LPMO9 enzymes characterized so far. Figure 2 shows a multiple-sequence alignment including the *Gt*LPMO9s. The catalytic domains of all four LPMOs contain the two characteristic copper-binding histidines (His1 and His86 in *Gt*LPMO9A-2), as well as a conserved tyrosine that occupies one of the axial positions in the copper coordination sphere (Tyr175 in *Gt*LPMO9A-2) (6).

*Gt*LPMO9A-2 and *Gt*LPMO9D have an additional C-terminal domain that starts with a region of low sequence complexity that might be a linker (see Fig. S2 in the supplemental material). We were not able to detect sequence similarity with known carbohydrate-binding domains (CBMs) or with other domains of known function. For more detailed sequence analysis of the C-terminal domains, see the supplemental material (including Fig. S2 to S4). Notably, the sequence alignments of Fig. S3 and S4 in the supplemental material show that the C-terminal domains of *Gt*LPMO9A-2 and *Gt*LPMO9D also occur in other LPMOs. Furthermore, the alignment shows that the C-terminal domain of *Gt*LPMO9A-2 has features not unlike those of well-known cellulose-binding domains.

As a first step toward mapping the ability of G. trabeum to oxidatively cleave plant polysaccharides, we have overexpressed and characterized *Gt*LPMO9A-2. This enzyme was selected as a representative for three (*Gt*LPMO9A-1, *Gt*LPMO9A-2, and *Gt*LPMO9B) of the five G. trabeum LPMOs that are closely related (Fig. 1) and that cluster in an area of the phylogenetic tree with little available functional data. Also, the coding sequence of *Gt*LPMO9A-2 has not been reported before. *Gt*LPMO9D was also considered interesting, but we were not able to express this enzyme in sufficient amounts.

### Physical properties of recombinant *Gt*LPMO9A-2.

*Gt*LPMO9A-2 was expressed in Pichia pastoris and purified to homogeneity by 3 steps of column chromatography (see Fig. S5 in the supplemental material). Purified *Gt*LPMO9A-2 appeared to have a significantly higher molecular mass (ca. 60 kDa) than that estimated from the amino acid sequence (36.0 kDa). Treatment with Endo H led to a small decrease in molecular mass (to approximately 57 kDa), indicating *N*-glycosylation of the protein. The remaining molecular mass difference suggests that the recombinant enzyme was also *O*-glycosylated, perhaps in the serine-rich linker region. Correct processing of the purified protein was confirmed by determination of N-terminal amino acid sequence of the purified enzyme, yielding HGYVDQVTIG, which is identical to the amino acid sequence deduced from the nucleotide sequence. As observed previously for LPMO9s expressed in P. pastoris, the N-terminal histidine was not methylated (14, 15, 51).

### Cellulolytic activity of recombinant *Gt*LPMO9A-2.

**FIG 3:**
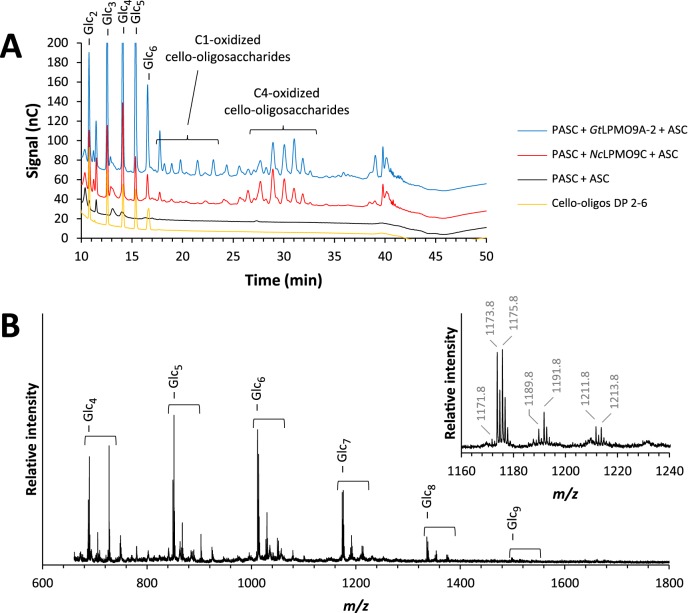
Products generated by *Gt*LPMO9A-2 or *Nc*LPMO9C on PASC. (A) HPAEC-PAD chromatograms showing cello-oligosaccharides released by *Gt*LPMO9A-2 (blue) and *Nc*LPMO9C (red) from PASC. Peaks were assigned based on previous assignments by Isaksen et al. (50); native cello-oligosaccharides are labeled as Glc_n_, where n is the degree of polymerization (DP). Note that it has recently been shown that C-4-oxidized products are unstable under these chromatographic conditions and that the peaks labeled C-4 are, in fact, diagnostic degradation products (34). The fractions of native products are high because C-4-oxidized products tend to lose the oxidized monosugar under these chromatographic conditions. (B) MALDI-TOF spectrum of cello-oligosaccharides released by *Gt*LPMO9A-2 from PASC, where the inset shows details for the heptamer ion cluster (sodium adducts only). Possible products in these clusters are the native Glc_7_ (*m/z* 1,175.8), the C-1-oxidized lactone, or C-4-oxidized ketoaldose (anhydrated species, *m/z* 1,173.8), the C-1-oxidized aldonic acid or C-4-oxidized gemdiol (hydrated species, *m/z* 1,191.8), and the sodium adduct of the aldonic acid sodium salt (*m/z* 1,213.8). The double-oxidized species corresponds to *m/z* 1,171.8 (anhydrated form, lactone-ketoaldose species), *m/z* 1,189.8 (hydrated form), and *m/z* 1,211.8 (the sodium salt of the sodium adduct). The presence of sodium salts is diagnostic for C-1 oxidation, since only this oxidation yields an aldonic acid. The strong signals for dehydrated oxidized species in MALDI-TOF are diagnostic for C-4 oxidation. The 4-keto to gemdiol equilibrium is less skewed toward the hydrated form than the lactone-aldonic acid equilibrium, and, besides, C-4-oxidized products are more efficiently dehydrated during spotting on MALDI sample plates (see Forsberg et al. [66] for further discussion). Experiments with Avicel yielded similar product patterns (data not shown).

*Gt*LPMO9A-2 was active on phosphoric acid-swollen cellulose (PASC) (Fig. 3), similar to all LPMO9s characterized so far (52), and Avicel (data not shown). HPAEC analysis showed diagnostic product patterns that reflect a mixed C-1/C-4 oxidation pattern, similar to the phylogenetically closest characterized LPMO (Fig. 1), *Nc*LPMO9M (NCU-07898 [49]). The type of electron donor, ascorbic acid or DTT, had no effect on the product profile of the LPMO (see Fig. S6 in the supplemental material), but the reaction was slower when using DTT. *Gt*LPMO9A-2 was inactive on shorter cello-oligosaccharides, such as cellopentaose (see Fig. S7A in the supplemental material) and cellohexaose (not shown), compared to *Nc*LPMO9C. Neither *Gt*LPMO9A-2 nor *Nc*LPMO9C showed activity on XG7 (not shown). Interestingly, *Gt*LPMO9A-2 was active on carboxymethyl cellulose (CMC) (see Fig. S8 in the supplemental material), suggesting that the enzyme can act on soluble polymeric β-glucans and is not restricted by substitutions on the d-glucose backbone.

### Hemicellulolytic activity of recombinant *Gt*LPMO9A-2.

**FIG 4:**
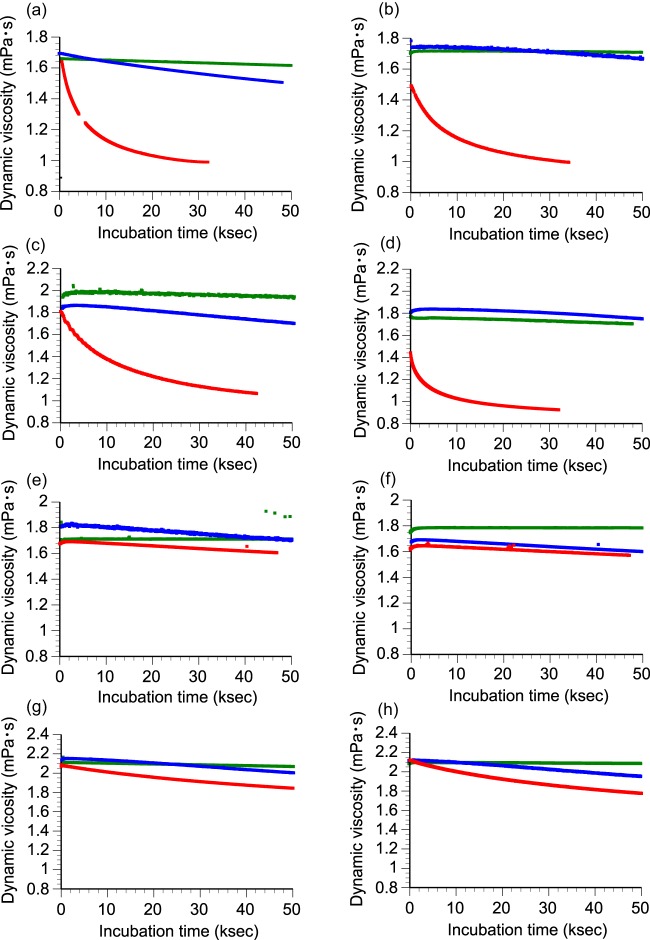
Assessing LPMO activity on hemicelluloses with dynamic viscosity experiments. *Gt*LPMO9A-2 (a, c, e, and g) or *Nc*LPMO9C (b, d, f, and h) was incubated with 0.15% (wt/vol) xyloglucan (a and b), 0.05% (wt/vol) glucomannan (c and d), 0.2% (wt/vol) arabinoxylan (e and f), and 0.5% (wt/vol) carboxymethyl cellulose (g and h) in the presence (red lines) or absence (green lines) of DTT as a reducing agent. Reactions with only DTT and no LPMO are shown by blue lines.

The activity of *Gt*LPMO9A-2 against hemicellulosic substrates was assayed using dynamic viscosity analysis, using *Nc*LPMO9C, which has known activity toward several β-1,4-glucans (13), as a positive control. As expected, significant decreases in viscosity were observed when xyloglucan (XG; Fig. 4b) or glucomannan (GM; Fig. 4d) was incubated with *Nc*LPMO9C, whereas no significant change in viscosity was detected in the reaction with arabinoxylan (Fig. 4f). Similarly to *Nc*LPMO9C, *Gt*LPMO9A-2 also caused a decrease in viscosity in the reactions with XG (Fig. 4a) and GM (Fig. 4c) but not with arabinoxylan (Fig. 4e). Both enzymes also reduced slightly the viscosity in reactions with CMC (Fig. 4g and h). The dynamic viscosity remained unchanged in reactions without the LPMO or an electron donor, showing that the observed effects are due to oxidative cleavage by the LPMOs and not to, e.g., contaminating hydrolases. The fact that treatment with the LPMOs led to a drop in viscosity in the presence (but not in the absence) of an electron donor clearly indicates that both enzymes have an endo-type action on XG and GM.

Comparative studies revealed that *Gt*LPMO9A-2 reduced the viscosity of XG almost 2-fold faster than *Nc*LPMO9C, whereas the difference in final viscosity was negligible (Table 2). The viscosity data indicated that the Gloeophyllum enzyme was slightly less active than the Neurospora enzyme on GM, both in terms of initial rate and final viscosity. Both *Gt*LPMO9A-2 and *Nc*LPMO9C were 5- to 10-fold slower in reducing the viscosity of a CMC solution (0.5% [wt/vol]) than with XG (0.15% [wt/vol]).

**TABLE 2:** Parameters obtained after fitting the dynamic viscosity curves^*a*^

Substrate	Enzyme	Fit	Parameter			*R*^2^^*b*^	IDR (mP · ks^−1^)^*c*^
			*a* (mP)	*b* (ks^−1^)	*c* (mP)		
XG	*Gt*LPMO9A-2	Exp.	0.5748	−0.1514	0.9812	0.9927	0.0870
	*Nc*LPMO9C	Exp.	0.4633	−0.1067	0.9947	0.9950	0.0495
GM	*Gt*LPMO9A-2	Exp.	0.7115	−0.07416	1.0496	0.9966	0.0528
	*Nc*LPMO9C	Exp.	0.3907	−0.1537	0.9367	0.9832	0.0600
WAX	GtLPMO9A-2	Lin.	−0.001454	1.7106		0.9936	0.001454
	*Nc*LPMO9C	Lin.	−0.001654	1.663		0.9731	0.001653
CMC	*Gt*LPMO9A-2	Exp.	0.3713	−0.01999	1.7082	0.9998	0.0074
	*Nc*LPMO9C	Exp.	0.4455	−0.02732	1.6661	0.9991	0.0122

a The correlation between dynamic viscosity and time (data from Fig. 4>) was fitted to an exponential (Exp.) curve (*y* = *a* × e^[*b* × *x*]^ + *c*) or to a linear (Lin.) equation (*y* = *a* × *x* + *b*), where *y* is the dynamic viscosity (in millipoise), *x* is the time (in kiloseconds), and *a*, *b*, and *c* are constants. The constant *c* is the final viscosity of substrate after completion of LPMO9 action.

b The high *R*^2^ values (>0.97) reflect a good fit of the model.

c The initial depolymerization rate (IDR) was calculated as the first derivative for the formula at *x* = 0.

We used HPAEC-PAD and MALDI-TOF MS to analyze water-soluble oligosaccharides potentially released by *Gt*LPMO9A-2 from a selection of hemicellulosic substrates. No products could be detected upon incubating the LPMO (plus electron donor) with ivory nut mannan (a linear β-1,4-linked mannan), wheat arabinoxylan, oat spelt xylan, beech wood xylan, and birchwood xylan (heteropolymers with β-1,4-linked xylan backbone) or mixed linked β-glucan (with a β-1,3 linkage at every third linkage in the β-1,4-linked glucan backbone) (data not shown). The data for XG and GM are discussed below.

### Identification of oligosaccharides released from xyloglucan and glucomannan.

**FIG 5:**
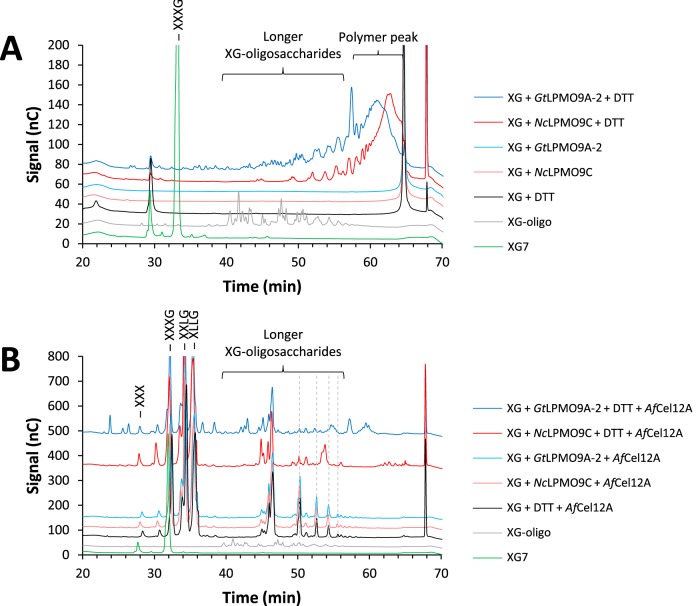
HPAEC-PAD analysis of reaction products generated from xyloglucan (XG) by *Gt*LPMO9A-2 and *Nc*LPMO9C in the dynamic viscosity experiments. Samples were taken after 16 h of incubation, and the chromatograms show the product profiles before (A) and after (B) a subsequent treatment with *Af*Cel12A. Green lines, XG7 standard (XXXG); gray lines, XG-oligomer standard (this is a mixture of shorter XG oligomers with DP in the range of 14 to 27); black lines, XG incubated with DTT only; red lines, products generated from XG with *Nc*LPMO9C in the presence (dark red) or absence (light red) of DTT; blue lines, products generated from XG with *Gt*LPMO9A-2 in the presence (dark blue) or absence (light blue) of DTT. Gray dashed lines indicate oligosaccharides, the concentrations of which were lower as a result of LPMO activity. The reaction conditions are specified in Materials and Methods. Note that Fig. 7 provides an even clearer example of the difference between *Nc*LPMO9C and *Gt*LPMO9A-2 with respect to the activity on xyloglucan.

To confirm LPMO action on hemicelluloses, which eventually would lead to the formation of oligosaccharides, we subjected the endpoint samples from the dynamic viscosity experiments to HPAEC-PAD analysis. The HPAEC-PAD profiles showed that *Gt*LPMO9A-2 released a broad range of xyloglucan oligosaccharides eluting from 25 to 60 min, whereas the major products generated by *Nc*LPMO9C eluted between 45 and 60 min (Fig. 5A). No products could be detected when either of the LPMOs or the reducing agent was incubated with the substrate alone. To simplify the product profiles, we treated the samples with *Af*Cel12A, an XG-active endoglucanase, which depolymerizes XG into its repeating oligomeric units by cleaving next to nonsubstituted glucose units. The known dominant products of *Af*Cel12A are XXXG, XXLG/XLXG, XLLG (G, glucose; X, glucose carrying a xylose substitution at C-6; L, X with a further galactose appended to the C-2 of the xylose). Note that the basic repeating unit in tamarind xyloglucan is XXXG carrying galactosylations at various positions (13, 53). In addition, the tamarind xyloglucan was partially arabinosylated, carrying 0.27 arabinosyl units per repeating (XXXG) unit (compositional data provided by the manufacturer, Megazyme).

The resulting chromatograms (Fig. 5B) are dominated by standard (nonoxidized) XG breakdown products that were released by the endoglucanase from the still mainly polymeric XG remaining after the LPMO treatment. The dominating products with a four-glucan backbone (XXXG fragments with various degrees of galactosylation) elute between 30 and 40 min, whereas less-abundant longer products elute between 45 and 55 min in discrete peaks (Fig. 5B, black lines). The less-abundant longer products are likely to be a variety of oligomers of degree of polymerization (DP) 12 to 18, some of which contain arabinosyl units (54). Interestingly, some of these oligomeric products (marked with gray dashed lines in Fig. 5B) were absent in samples that had been treated with LPMO plus reductant (compare reactions without [light blue and red lines] and with [dark blue and red lines] reducing agent in Fig. 5B). Next to these standard products, the product profiles from reaction mixtures that had been pretreated with an active LPMO showed additional peaks, and the patterns of these additional peaks reveal differences between the two enzymes. Endoglucanase treatment of the product mixture generated by *Gt*LPMO9A-2 yielded a wide variety of additional products, with elution times ranging from 20 to 60 min and including products eluting earlier than XXXG (XG7) (Fig. 5B, green lines). In contrast, endoglucanase treatment of *Nc*LPMO9C-generated material yielded a less-varied collection of additional products, which almost exclusively eluted at 45 min or later and which included a cluster of products eluting at 61 to 66 min that is lacking from the reaction mixtures with *Gt*LPMO9A-2. The product profile obtained here with *Nc*LPMO9C is similar to what has been observed before for this C-4-oxidizing enzyme (13), whereas the product profile for *Gt*LPMO9A-2 is clearly different. Based on data presented by Bennati-Granier et al. (14), the peak eluting at 43 min that is unique for *Gt*LPMO9A-2 might be C-1-oxidized XG7.

HPAEC-PAD chromatograms for the reactions with konjac GM showed clear effects of *Nc*LPMO9C, whereas the effects of *Gt*LPMO9A-2 on GM were less pronounced (see Fig. S9A in the supplemental material). Treatment of GM with *Nc*LPMO9C led to a clear shift in the polymer peak, and small amounts of oligomeric products were also observed. For the reaction with *Gt*LPMO9A-2, only a minor shift in the polymeric peak was observed. Subsequent endoglucanase treatment with *Ta*Cel5A led to the accumulation of chromatographically distinct products in the case of *Nc*LPMO9C, while similar products were not observed after the combined action of *Gt*LPMO9A-2 and *Ta*Cel5A (see Fig. S9B).

**FIG 6:**
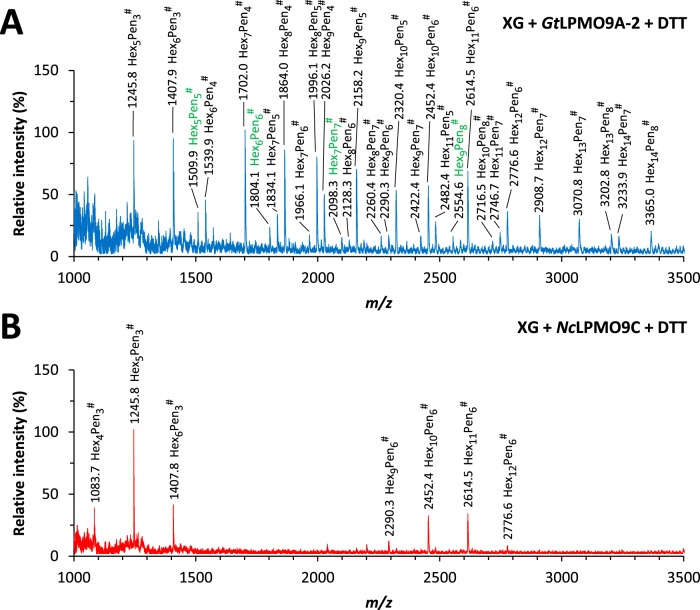
MALDI-TOF MS analysis of the reaction products generated by *Gt*LPMO9A-2 (A) and *Nc*LPMO9C (B) during the dynamic viscosity experiments with tamarind xyloglucan (XG) in the presence of reducing agent (DTT). The samples analyzed were the endpoint samples (16 h). The reaction conditions are specified in Materials and Methods and were similar to the conditions used for generating Fig. 5. *Gt*LPMO9A-2 generated a wide range of oligosaccharides with all possible combination of hexose (Hex) and pentose (Pen) units, whereas *Nc*LPMO9C generated clusters of oligosaccharides with the number of pentose residues being a multiple of three. In both spectra, oxidized species (−2, compared with the weight of native species) are marked with #; species with masses indicating arabinosylation are labeled green (see the text). Note that most labeled peaks in fact are a cluster of signals; see Fig. S10 in the supplemental material and text. All labeled species are Na^+^ adducts. No species were detected below *m/z* 1,000.

MALDI-TOF MS analysis of the reaction mixtures obtained after the dynamic viscosity experiments confirmed major differences in the product profiles generated by *Gt*LPMO9A-2 and *Nc*LPMO9C (Fig. 6). While *Nc*LPMO9C released clusters of oxidized xyloglucan fragments, with the number of xylose residues being a multiple of three, *Gt*LPMO9A-2 released a much broader range of oxidized oligosaccharides. The results for *Nc*LPMO9C are in accordance with previous work (13) showing that the enzyme cleaves XG at the nonreducing end of the nonsubstituted glucosyl residue in the (XXXG)_n_ backbone of xyloglucan, forming C-4-oxidized XG fragments. On the other hand, the data for *Gt*LPMO9A-2 suggest that this LPMO can cleave almost anywhere in the XG chain, since reactions with this enzyme yield oxidized XG fragments with a number of xylose residues not being only a multiple of three. The addition of LPMO in the absence of reducing agent did not have an impact on the product profiles obtained from XG after endoglucanase treatment. This indicates that the *Gt*LPMO9A-2 and *Nc*LPMO9C enzyme preparations were not contaminated with background activity of an enzyme that would cleave off xylosyl units of XG, and that all XG fragments in Fig. 6 were products of LPMO treatment. Upon treatment of GM with *Gt*LPMO9A-2, we could not detect oxidized oligosaccharides with MALDI-TOF MS.

A closer inspection of the data in Fig. 6 (see Fig. S10 in the supplemental material) showed that the labeled signals correspond to clusters containing signals for the native, the nonhydrated oxidized, and the hydrated oxidized oligosaccharide, with the native and nonhydrated oxidized signals dominating. Signals for the sodium salt of the aldonic acid and double-oxidized species, which would be indicative of C-1 oxidation, were not observed. Although it is not certain that oxidized xyloglucan oligosaccharides behave like oxidized cello-oligomers under these analytical conditions (see Fig. 3 legend), the data suggest that C-4 oxidation dominated during the degradation of XG.

In addition to the expected masses originating from galactosylated XG fragments, the MALDI-TOF spectra for the reactions with *Gt*LPMO9A-2 contained a few species with more than three pentose units per repeating unit (4 hexoses) and hence are predicted to carry an arabinosylated X-unit (Fig. 6) (54). Since the arabinosyl and xylosyl groups (both pentoses) cannot be distinguished by MS, we cannot exclude that some of the other fragments generated by *Gt*LPMO9A-2 also contain an arabinosyl group replacing a xylosyl unit (see proposed structures by Niemann et al. [54]).

The chromatogram in Fig. 5B shows that incubation of the LPMO-treated xyloglucan sample with *Af*Cel12A resulted in the production of shorter xyloglucan oligosaccharides, producing mostly native oligosaccharides with DP of 7 to 9 and a wide variety of putatively oxidized products, particularly in the case of *Gt*LPMO9A-2 (Fig. 5B). MALDI-TOF MS (results not shown) showed that many of the higher-DP species visible in Fig. 5B and 6A were no longer present after combined endoglucanase-LPMO action. The combined action of *Gt*LPMO9A-2 and *Af*Cel12A on XG resulted in the formation of shorter fragments (in particular, *m/z* 494.1 [oxidized XG/GX or L] and 791.4 [native XXG/XGX/GXX or XL/LX]). These shorter native species may correspond to the compounds eluting before the XG7 peak, at 20 to 30 min, in Fig. 5B. It is noteworthy that the native species observed with HPAEC may also originate from C-4-oxidized products, as it has recently been shown that C-4-oxidized products are unstable and tend to lose the oxidized monosugar under the chromatographic conditions used here (34).

### LPMO activity on complex substrates.

**FIG 7:**
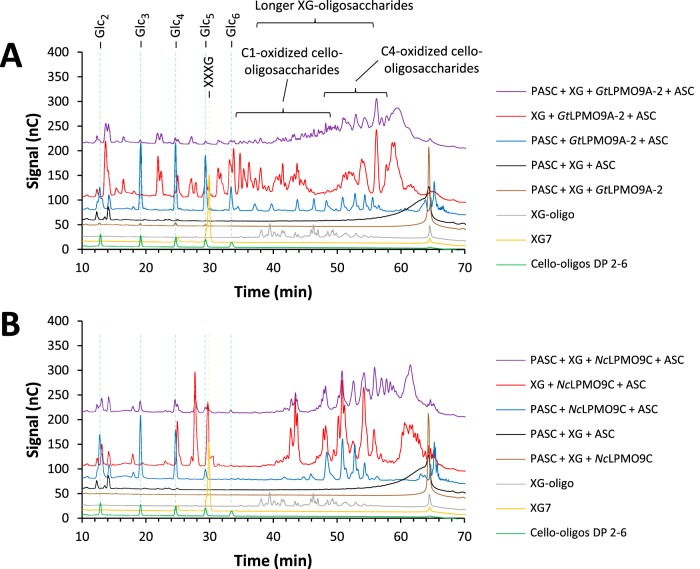
HPAEC-PAD analysis of the reaction products generated by *Gt*LPMO9A-2 (A) or *Nc*LPMO9C (B) on xyloglucan-coated PASC. Green lines, native cello-oligosaccharides with DP 2 to 6; yellow lines, XG7 standard (XXXG); gray lines, XG-oligomer standard with DP 14 to 27; brown lines, mixture of PASC and XG incubated with LPMO only; black lines, mixture of PASC and XG incubated with ascorbic acid (ASC) only; blue lines, products generated from PASC with LPMO in the presence of ASC; red lines, products generated from XG with LPMO in the presence of ASC; purple lines, products generated from xyloglucan-coated PASC with LPMO in the presence of ASC. The reaction conditions are specified in Materials and Methods. Note that the chromatographic conditions were similar to those used for the experiment whose results are shown in Fig. 5 but different from those used for the experiment whose results are shown in Fig. 3. This explains why the retention times of cello-oligomers differ between the data in Fig. 3 and 5.

In order to evaluate possible additional differences between *Gt*LPMO9A-2 and *Nc*LPMO9C and to study LPMO action on more natural substrates, we then carried out experiments with copolymeric substrates generated by mixing xyloglucan or glucomannan with PASC, using ascorbic acid as a reducing agent (Fig. 7; note that the reaction is faster when using ascorbic acid, explaining the apparent differences between the data in Fig. 7 and 5A). These experiments were inspired by the findings by Frommhagen et al. (17), who demonstrated LPMO activity on xylan, but only if the xylan was grafted onto cellulose. In general, hemicellulose coating hindered LPMO activity on cellulose, reducing the amount of oxidized cello-oligosaccharides released (compare blue and purple lines in Fig. 7 and in Fig. S10 in the supplemental material). Interestingly, coating XG on cellulose also seemed to have a negative effect on the activity of both LPMOs toward XG itself (compare red and purple lines in Fig. 7). Experiments with GM-coated PASC and *Gt*LPMO9A-2 showed that coating with GM completely abolished *Gt*LPMO9A-2 activity on PASC, whereas soluble GM products were not detected by the HPAEC-PAD analysis (see Fig. S11 in the supplemental material). Interestingly, control reactions with *Nc*LPMO9C, with much higher activity on GM, showed that this LPMO degraded the GM and that, consequently, the degradation of PASC was still observed after coating with GM. We carried out several experiments with birchwood xylan, alone or mixed with PASC, without detecting any activity on xylan.

## DISCUSSION

The present data show that *Gt*LPMO9A-2 is a C-1/C-4-oxidizing LPMO with high activity on cellulose and xyloglucan, negligible activity on cellodextrins, and minor activity on glucomannan. The enzyme has an exceptional capability to cleave any Glc-Glc bond in xyloglucan, regardless of substitutions, thus functionally expanding the arsenal of hemicellulolytic LPMOs so far described (13, 14, 16, 17). Xylosyl substitutions at the C-6 position in XG seemed to be less inhibitory than carboxymethyl substitutions in CMC, which may be located at the C-2, C-3, or C-6 position on the glucan backbone. The fact that *Gt*LPMO9A-2 was not active on cello-oligosaccharides, nor on mixed-linked glucan, suggests that *Gt*LPMO9A-2, in contrast to *Nc*LPMO9C, requires longer stretches of β-1,4-glucosidic linkages in the polysaccharide backbone for substrate recognition. Perhaps, the minor depolymerizing activity that we observed with konjac glucomannan reflects the occasional presence of longer stretches of β-1,4-linked glucose units. The fact that *Gt*LPMO9A-2 was inactive toward XG7 (consisting of a cellotetraose backbone), cellopentaose, and cellohexaose may be taken to imply that the enzyme requires at least seven glucosyl units for productive substrate binding.

So far, only three xyloglucan-active LPMOs have been studied in detail, *Nc*LPMO9C from Neurospora crassa (13), *Pa*LPMO9H from Podospora anserina (14), and *An*3046 from Aspergillus nidulans (16). All these LPMOs cleave xyloglucan next to the nonsubstituted glucosyl residue (i.e., G-unit), yielding a clustered product profile on MALDI-TOF MS (as seen in Fig. 6B). In contrast, the oxidized products released by *Gt*LPMO9A-2 had a wide variety of *m/z* values (Fig. 6A), showing that *Gt*LPMO9A-2 activity is unaffected by substitution of the glycosyl units at the C-6 position and that *Gt*LPMO9A-2 can cleave everywhere in the β-glucan main chain. These observations are further supported by the wide range of products observed upon the combined action of *Gt*LPMO9A-2 and *Af*Cel12A (which only cleaves at nonsubstituted Gs), including substituted products with dimeric or trimeric backbones that only can emerge upon cleavage between two substituted main-chain sugars.

The oxidative regioselectivities of xyloglucan-active LPMOs on cellulose show that all variants occur, C-1 (*An*3046; literature data are not conclusive, and C-1/C-4 activity is also possible), C-4 (*Nc*LPMO9C), and C-1/C-4 (*Pa*LPMO9H and *Gt*LPMO9A-2). However, there are little data on the oxidative regioselectivity of these enzymes on xyloglucan. Our own previous data for *Nc*LPMO9C and the present data for *Gt*LPMO9A-2 indicate that these enzymes almost exclusively oxidize C-4 when acting on XG.

The sequence alignment of Fig. 2 shows that *Gt*LPMO9A-2 has an extended L2 loop (compared to *Nc*LPMO9C and *Pa*LPMO9H), while it lacks the L3 loop and also has a deletion (relative to *Nc*LPMO9C and *Pa*LPMO9H) in the so-called LC loop, very close to a conserved surface-exposed aromatic residue (Tyr204 in *Nc*LPMO9C and Tyr215 in *Gt*LPMO9A-2). The same applies to *An*3046, although this was not noted by the authors (16). The L3 loop has been proposed to be a structural determinant of xyloglucan activity (14), and a recent nuclear magnetic resonance (NMR) study of enzyme-substrate interactions in *Nc*LPMO9C showed that the L3 loop indeed interacts with xyloglucan (55). The present data and data for *An*3046 show that xyloglucan cleavage can also be achieved by LPMOs lacking the L3 loop. The extension of the L2 loop may compensate for the deletion of the neighboring L3 loop, and it is interesting to note that the extension and deletion are of the same length, 14 amino acids. Lack of the L3 loop and the seemingly correlated deletion near Tyr204/215 may have effects on the specificity of xyloglucan cleavage and/or the ability to cleave shorter substrates. For example, only the two xyloglucan-active LPMOs with the L3 loop and with no deletion in the LC loop, i.e., *Nc*LPMO9C and *Pa*LPMO9H, have been shown to cleave soluble cellodextrins (14, 50).

Viscosity measurements have occasionally been used to assess the activity of endoglucanases on water-soluble polysaccharides (56–58). Here, we show that dynamic viscosity measurements provide an alternative and sensitive method for assessing and comparing LPMO activity on water-soluble polysaccharides. This simple method proved to be a good choice for both characterizing substrate specificity and comparing depolymerization rates. In fact, by relying solely on the standard methods (HPAEC-PAD and MALDI-TOF MS) that are based on monitoring released oligomeric products, we would have overlooked the low activity of *Gt*LPMO9A-2 on glucomannan. These findings show the need for using complementary methods, including methods capable of monitoring the polymeric fraction, when characterizing LPMOs.

Furthermore, while the quantification of LPMO activity is complex, due to product heterogeneity and lack of product solubility, the dynamic viscosity measurements provide a direct measurement of the number of cuts introduced in the polysaccharide substrate. This method is independent of oxidative regioselectivity and the cleavage pattern. In the present study, the dynamic viscosity experiments showed that *Nc*LPMO9C is more active on glucomannan than *Gt*LPMO9A-2, whereas *Gt*LPMO9A-2 depolymerized xyloglucan at a higher rate. The higher activity of *Gt*LPMO9A-2 on xyloglucan might be due to its broader cleavage specificity, which implies that there are more productive binding sites on the substrate.

The experiments with copolymeric substrates yielded somewhat surprising results. We show that hemicellulose coating of PASC reduces LPMO activity on PASC dramatically. This provides a rationale for hemicellulolytic activity among LMPOs, as exemplified by the studies with glucomannan. *Nc*LPMO9C, with its glucomannan activity, was able to degrade GM-coated PASC (degrading both GM and PASC), whereas *Gt*LPMO9A-2, with only very weak GM activity, could not degrade the mixture of these two polymers. On the other hand, in mixtures of cellulose and XG, the activity on both polymers was strongly reduced for both tested LPMOs (see below for further discussion).

To date, there is limited information on LPMOs from brown-rot fungi. Vanden Wymelenberg et al. showed low expression levels of LPMO genes in the brown-rot fungus Postia placenta even when cultivated on aspen wood (59). The expression of LPMOs was undetectable in proteome analyses for the brown-rot fungi Fomitopsis pinicola and Wolfiporia cocos growing on aspen wood (60). These results indicate that LPMOs may not constitute a major enzyme component in the wood degradation system of brown-rot fungi. However, Jung et al. recently reported that saccharification of oak and kenaf pretreated with popping was promoted by a homologue of *Gt*LPMO9B, suggesting the importance of this enzyme in the enzymatic degradation of plant biomass by brown-rot fungi (32). Moreover, we were able to amplify all G. trabeum LPMOs from mRNA, meaning that the genes are expressed, and we showed that *Gt*LPMO9A-2 is active on biomass.

Brown-rot fungi, such as G. trabeum, preferentially invade softwood. In softwood, the main hemicelluloses are glucomannan and glucuronoxylan, which occur in the secondary cell wall. The secondary cell wall is surrounded by a primary cell wall, where the most abundant hemicelluloses are xyloglucan and pectin (61–63). The genome of G. trabeum encodes sets of CAZymes that target xyloglucan (glycoside hydrolase 12s [GH12s] and GH74) and pectin (10 GH28s, GH43s, 2 CE8s, and CE15), besides cellulose (e.g., GH3s, GH5s, and GH12s), glucomannan (GH3s and GH5s), and xylan (3 GH10s, GH43s, CE1, and 6 CE16s) (29). Due to its substrate promiscuity, *Gt*LPMO9A-2 might play a role in the depolymerization of not only cellulose but also xyloglucan during biomass degradation. Perhaps, the enzyme has additional capabilities that remain undiscovered, such as activities on natural copolymeric plant cell walls. It might be of interest to test other electron donors, since there are indications that the electron donor affects LPMO performance, including substrate specificity (11, 31). By degrading xyloglucan, *Gt*LPMO9A-2 may loosen the adhesion between cellulose bundles, thus creating access for various CAZymes. The fact that *Gt*LPMO9A-2 (and also *Nc*LPMO9C) acted preferentially on free XG compared to cellulose-bound XG suggests that *Gt*LPMO9A-2 may preferentially cut XG chains that tether cellulose fibers together, as opposed to XG chains that adhere to cellulose fiber surfaces.

It is well known that wood strength rapidly decreases at the initial stage of brown rot even before the loss of weight in wood (64). As the fungus invades the wood cells from the lumen, depolymerization of cell wall components using a low-molecular-weight decay system facilitates enzyme penetration into the cell wall and enables enzymatic degradation of the outer primary cell wall concurrent with the inner secondary cell wall layers (22). The rapid decrease in cellulose DP throughout the wood cell wall and the degradation of the primary cell wall are likely to be primary causes of strength reduction at this decay stage (22). It is tempting to speculate that *Gt*LPMO9A-2 plays a role in this process, i.e., primary cell wall degradation at the initial stage of brown rot.

## Supplementary Material

Supplemental material
