# Supplementary material for: A Lytic Polysaccharide Monooxygenase with Broad Xyloglucan Specificity from the Brown-Rot Fungus Gloeophyllum trabeum and Its Action on Cellulose-Xyloglucan Complexes
Source: Appl Environ Microbiol. 2016 Oct 27;82(22):6557–72. doi: 10.1128/AEM.01768-16 (PMC5086550; doi:10.1128/AEM.01768-16)
Supplement: Supplemental material [file supp_82_22_6557__index.html]

Supplemental material 

# A Lytic Polysaccharide Monooxygenase with Broad Xyloglucan Specificity from the Brown-Rot Fungus Gloeophyllum trabeum and Its Action on Cellulose-Xyloglucan Complexes

## Supplemental material

- Supplemental file 1 -

  Splicing variants of the gene encoding *Gt*LPMO9A (Fig. S1); sequences of the C-terminal extensions of *Gt*LPMO9A-2 and *Gt*LPMO9D (Fig. S2); multiple sequence alignment of the C-terminal domain of *Gt*LPMO9A­-2 with homologous domains (Fig. S3); multiple sequence alignment of the C terminus of *Gt*LPMO9D with similar C termini of LPMOs (Fig. S4); SDS-PAGE of recombinant *Gt*LPMO9A-2 produced in *Pichia pastoris* (Fig. S5); comparison of the product profile of *Gt*LPMO9A-2 on PASC with ascorbic acid or DTT as electron donor (Fig. S6); products generated by *Gt*LPMO9A-2 or *Nc*LPMO9C from oligosaccharides (Fig. S7); products generated by *Gt*LPMO9A-2 or *Nc*LPMO9C from carboxymethylcellulose (Fig. S8); HPAEC-­PAD analysis of reaction products generated from konjac glucomannan by *Gt*LPMO9A-2 or *Nc*LPMO9C in the dynamic viscosity experiments (Fig. S9); details of the Hex6Pen3 and Hex7Pen5/Hex8Pen4 ion clusters in the MALDI-­ToF spectrum for the end-­point sample from the dynamic viscosity analysis where *Gt*LPMO9A-2 or *Nc*LPMO9C reacted with tamarind xyloglucan in the presence of reducing agent (Fig. S10); HPAEC-PAD analysis of reaction products generated by *Gt*LPMO9A-2 or *Nc*LPMO9C on glucomannan-coated PASC (Fig. S11).

  PDF, 1.1M
