# Supplementary material for: A Lytic Polysaccharide Monooxygenase with Broad Xyloglucan Specificity from the Brown-Rot Fungus Gloeophyllum trabeum and Its Action on Cellulose-Xyloglucan Complexes
Source: Appl Environ Microbiol. 2016 Oct 27;82(22):6557–72. doi: 10.1128/AEM.01768-16 (PMC5086550; doi:10.1128/AEM.01768-16)
Supplement: Supplemental material [file AEM.01768-16_zam022167501so1.pdf]

Characterization of an LPMO from the brown-rot fungus *Gloeophyllum trabeum* with broad xyloglucan specificity, and its action on cellulose-xyloglucan complexes – Supplemental material

Authors: Yuka Kojima[1,*], Anikó Várnai[2,*], Takuya Ishida[3], Naoki Sunagawa[3], Dejan M. Petrovic[2], Kiyohiko Igarashi[3,4,], Jody Jellison[5], Barry Goodell[6], Gry Alfredsen[7], Bjørge Westereng[2], Vincent G.H. Eijsink[2,#], Makoto Yoshida[1,#]

Running title (max. 56 char): Enzymatic properties of a brown-rot LPMO

Section 1. Alternative splicing of the gene encoding GtLPMO9A (Fig. S1).

```
ATGTTCCGTGCCCAATCCTTCCTACCCGTCCTTGCTTTGGTTCTGCGGGTCGCTGCCCATGGATATGTTG
ATCAAGTCACGATAGGCGGACAAGTTTACACCGGATATCAGCCCTACCAGGACCCATACGAAAGCCCCGT
CCCCCAGCGTATTGAGAGAGCTATTCCAGGAAATGGGCCCGTCGAAGATCTCACACTCCTTGAGTGAGTG
AGTTGTTCAATGGGTACCTGAAGAGACGTACTGACTGTCACTCGCCGTGAAAGCATCCAATGTAATGGCT
CAGGGGGCTCCGGTACGAAGCCCGCCGCTCTCATAGCTTCAGCAGCCGCTGGGGATGAGATTGCCTTCCA
CTGGACTACATGGCCCAGTTCGCATGTCGTAAGTCACAAACGCTACGCAACTCATATAGCTCCCCATAAT
TTACCGCCAACAGGGCCCAGTCATTACTTACATGGGGAAAGTGCCTAGCAACACCGATATCACCAGCTAT
TCCCCCACTGGCTCCGACGTCATTTGGTTCAAGATTGACGAAGCAGGTTACGAGAACGGCAAGTGGGCCG
CTACGGATATCATGTCGGCACAGAATAGCACTTGGACTGTGACCATACCGAAGGCGCTCGCTCCGGGACA
GTACATTGTCCGTCATGAAATGTGAGAGATCGAAGGTTTTGCTGTATCGACCGGACCACTCAGCTCCATC
TTAAGAATCGCCCTGCACCAAGCTGAGACCTATCCCGGTGCTCAGTTTTACCCTGACTGCTTCCAAGTCC
AAGTCACCGGCCCTGGTACGGAGACACCCACATCTCAGGCTCTCGTGTCCTTCCCAGGCGGCTATACTCC
CACCACTCCCGGCATCACCTTCAACGTCTATAGCGGTAAGCACAGAGGTGACGTCATTAGACAAATCCTA
ACACAAATAAATTCATAGGTAGTATTACTTCATACCCCATCCCCGGTCCGCCTGTCTGGACGAGCAATGA
GGCTTTCTCCGGCGGGTCTTCCTCATCTGCTGCAGCCAGCAGCACGGCAGTCGCCTCTTCGACCGCTGAC
TCCTCATCCTCTGCGGCCGCCACCCAGTCCTCCTCCGCCGCCGCTTCGGGCTCCGCAGCACCCTCTAGCT
CTGCTATAGGTACGAGCACGGCTAGCTCCGCTGCCGCTAGCGGGACCGCCATCGTCGACGCGAACACCTG
CATGAACAGTGCGTAGTGTCTTCTACGCGTTATAAGGCACTTTACTGATTTTGGCGGCCTTTCCAGATTA
CAACAAGTGCATCGACGCCGGCCAGCCCGACCCTGACTGGAGCGGCTGCACCGCGACTAAAGATGCCTGC
CTGGCTGGCGCGACGTACCAGCGACTTGCTCGTTCTGGTACCCTGGGACGTCTCTCTTTCTAA
```

Figure S1. Splicing variants of the gene encoding *Gt*LPMO9A. Black colored regions indicate exons. Red colored regions indicate introns. The green colored region is an exon in the splicing variant that encodes *Gt*LPMO9A-1 but an intron in the splicing variant that encodes *Gt*LPMO9A-2. The stop codons for *Gt*LPMO9A-1 (TAG) and *Gt*LPMO9A-2 (TAA) are boxed.

Section 2. Sequence analysis of the C-terminal domains of GtLPMO9A-2 and GtLPMO9D (Figs. S2-S4 + additional text).

Fig. S2 shows the sequence of the C-terminal extensions of *Gt*LPMO9A-2 and *Gt*LPMO9D. These start with a region of low sequence complexity, followed by a domain of unknown function. BLAST searches with the C-terminus of *Gt*LPMO9A-2 domain revealed similarities with domains found in a few LPMOs and two GH131s (broad specificity exo-β-1,3/1,6-glucanases with endo-β-1,4-glucanase activity), all being uncharacterized proteins with predicted domains. A multiple sequence alignment (Fig. S3) revealed conspicuous features, namely four conserved Cys residues (Cys304, 311, 324, and 331) and three conserved aromatic residues (Tyr308, Trp321, Tyr337), which are features characteristic of CBM1s, i.e. small fungal, cellulose-specific carbohydrate-binding modules. The unknown domain ends with a short, positively charged motif containing 3-5 arginine/lysine (Fig.S3), which is atypical for CBM1s.

BLAST searches with the C-terminal domain of *Gt*LPMO9D revealed similarity with the C-terminal regions of a few basidiomycete LPMOs only (Fig. S4). This domain seems to consist of two positively charged, short sequences that are connected by 5 to 20 hydrophobic amino acids (Fig. S4). The first positively charged motif contains 3-5 lysines/arginines while the second motif contains 4-6 arginines/lysines with a conserved P+HΦSRΦM++ motif (where + is R or K, and Φ is a hydrophobic amino acid), which is likely to be an α-helix with a positive patch on one side.

Interestingly, none of the few LPMOs that have been characterized so far (or any cellulases and hemicellulases) carry a C-terminal domain with sequence homologous to that of *Gt*LPMO9A-2 or 9D. LPMO9s characterized so far carry 1) no CBM (some of the *N. crassa* LPMOs, *M. thermophila* LPMO9A, *T. terrestris* LPMO9E, *P. chrysosporium* LPMO9D), 2) the cellulose-binding CBM1 domain (e.g. some of the *N. crassa* and *P. anserina* LPMOs), or 3) an unknown C-terminal region (so far only *A. niger* LPMO AN3046; Jagadeeswaran et al. (1)).

GtLPMO9A-2 234 EAFSGGSSSSAAASSTAVASSTADSSSSAAATQSSSAAASGSAAPSSSAIGTSTASSAAA
294 SG**TAIVDANTCMNNYNKCIDAGQPDPDWSGCTATKDACLAGATYQRLARSGTLGRLSF**

GtLPMO9D 223 VSGDSGTVDGQGGSTSSAILSGGAAPTGTASGSTPAGTSQPSSTTGTGNAGAN**PSSGKCS**
283 **LKSRAAPTTSGNLSANYPRHFSRVMKRLLNDFQTTAHQW**

Figure S2. Sequences of the C-terminal extensions of *Gt*LPMO9A-2 and *Gt*LPMO9D. The C-terminal regions that were analyzed further by sequence alignment (see Figs. S3 & S4) are printed in bold and underlined.

```
ID
M7WKQ1        SQYTD-YTSCMRA-------------------------------------YNKCLDAHQP-
A0A061BI40    SQYTD-YTSCMRA-------------------------------------YNKCLDAHQP-
G0SWB0        SQYTD-YNSCMRA-------------------------------------YNKCLDAHQP-
A0A0K3CDZ6    SQYTD-YNSCMRA-------------------------------------YNKCLDAHQP-
A0A0D7AYH5    -CAKDGYNCCMDA-------------------------------------YNACAAKANSA
A0A0D7BPW0    AGLVD-CNTCMNS-------------------------------------FNQCISASQP-
GtLPMO9A-2    TAIVD-ANTCMNN-------------------------------------YNKCIDAGQP-
A0A0D7AX18    GGVQD-ANACMNT-------------------------------------YNKCIAQTQP-
D8PQJ8        TGIVD-ANACMNT-------------------------------------YNQCIAQSQP-
D8QGA8        AATGD-ANVCMNE-------------------------------------YNQCIAKSQP-
A0A0D7BLY4    SGQVD-ANVCMNS-------------------------------------YNKCIAQSQP-
A0A0D7BKR1    TGVVD-ANVCMND-------------------------------------YNKCIAASQP-
D8PXB3        KGIVD-ANCCMNGKFNIHLLVLLHCYMGRHFLPPESLRSHPRNPAFCQSKYNKCLAASQP-
R7RYA1        TAIVN-ANTCM--------------------------------------------------
                  :  . **                                       :* *.   :.
```

```
ID                                                             % Identity
M7WKQ1        -KNGGAADFSACQSMN--CQS---YQ----RMARRSARHAARH--       37.0%
A0A061BI40    -KNGGAADFSACQSMN--CQS---YQ----RMARRSARHAARH--       37.0%
G0SWB0        -KNGGAADFSACQSMN--CQS---YQ----RMARRSARHAARH--       39.1%
A0A0K3CDZ6    -KNGGAADFSACQSMN--CQS---YQ----RMARRSARHAARH--       39.1%
A0A0D7AYH5    ANNGGNVDFSACSSAKDTCVAG---------LARRHARQWSRQL-       34.0%
A0A0D7BPW0    -----SPDWTGCGATKDTCMSTCKY-------GKRSPSR------       56.8%
GtLPMO9A-2    -----DPDWSGCTATKDACLAGATYQ-----RLARSGTLGRLSF-      100.0%
A0A0D7AX18    -----NPDWTGCGATKDTCLSTAKY----MRRALKSGTLGRRLRA       58.9%
D8PQJ8        -----NPDWTGCGATKDSCLATATYNVNMAARAKRDGKFGRLLL-       64.3%
D8QGA8        -----NPDWTGCGATRDTCLSTARYNTNMAARAKRDGKFGRLLL-       55.4%
A0A0D7BLY4    -----NPDWTGCEATRTACLSNATYNFNMSQRARRDGKFGRLL--       61.8%
A0A0D7BKR1    -----NPDWTGCEGTKAICMQNATYNFNMINRLKRDGKFGRLLV-       60.7%
D8PXB3        -----NPDFTGCSAAKDTCMTGATYDYNMASRAKRDGKFGRLLL-       58.9%
R7RYA1        ---------SGCGSTKDTCMEGAVYE-----RVKRSVRFGRALA-       60.0%
                     *::.* . .  *              .
```

ID	Function	Organism
M7WKQ1	LPMO9	Rhodosporidium toruloides
A0A061BI40	LPMO9	Rhodosporidium toruloides
G0SWB0	LPMO9	Rhodosporidium toruloides
A0A0K3CDZ6	LPMO9	Rhodosporidium toruloides
A0A0D7AYH5	LPMO9	Cylindrobasidium torrendii
A0A0D7BPW0	LPMO9	Cylindrobasidium torrendii
**GtLPMO9A-2**	LPMO9	Gloeophyllum trabeum
A0A0D7AX18	LPMO10-Chitin-bd	Cylindrobasidium torrendii
D8PQJ8	LPMO9	Schizophyllum commune
D8QGA8	LPMO9	Schizophyllum commune
A0A0D7BLY4	LPMO9	Cylindrobasidium torrendii
A0A0D7BKR1	GH131	Cylindrobasidium torrendii
D8PXB3	putative GH131	Schizophyllum commune
R7RYA1	LPMO9	Stereum hirsutum

Figure S3. Multiple sequence alignment of the C-terminal domain of *Gt*LPMO9A-2 with homologous domains. This domain seems to contain four conserved cysteines (grey triangles),

three conserved aromatic residues (green diamonds), and a motif that is rich in positively charged amino acids. Aromatic amino acids (F, W, Y) are colored in green; positively charged amino acids (R, K) are colored in blue; negatively charged amino acids (D, E) are colored in red. The consensus symbols are indicated for the alignment excluding R7RYA1.

```
                                                                         α-helix?
ID                                                                       ———————→
A0A137QF73    SSPSK-CKLKKTAQPTASADASNSNSRRSLGDDSTSTGRSLNVKYYPKHISRVMRDLA--
A0A0D2M3B5    -TTGKTCKIQKRAVSNDEVDI---------------------FVVRPRHLSRIMRRLA--
A0A0C2XEH9    -APTKSCKIKKRAATSDEQNL---------------------VVRRPRHVSRIMRRLA--
A0A067SZR5    -PASKSCKIKKRAAAQDNE-----------------------LVVRPRHISRIMRRLA--
A0A0L6WMG1    -KSSGTCKLKRKASSSSLTVAA--------------------SIIRPRHLSRIMRGLIAN
W4K048        -SPSKQCNLKKRTAVNATKRD---------------------VASRPKHLSRIMRDLI--
I7DL47        -SPSKQCNLKKRAAGSATKRD---------------------VASRPKHLSRIMRDLI--
R7SQR2        -GSNAQCRLKKPSASNSSDL----------------------FVVRPRHLSRVMARLL--
A0A067P992    HHHTK-CSLKTSSPAATST-----------------------GALRPRHVSRVMRRMLVD
GtLPMO9D      PSSGK-CSLKSRAAPTTSGNL---------------------SANYPRHFSRVMKRLLND
A0A0C3C9X4    SKSGK-CQLQKQNT----------------------------VALYPRHFSRAMRRLL--
A8NLH8        -SSKKVCKLKKSKRSTTSD-----------------------SALYPRHFSRVMRRFI--
A0A0C9WJR9    SK-GKTCKLKKGKSASSSVVPSASAAAASATT----------TDMYPRHFSRVMRRLA--
B0DGU6        SK-GKSCKLKKGKTASSSVVPSASAAAASATT----------TDMYPRHFSRVMRRLA--
B0CQE5        TKS-KSCKLRQTKSASSSALSATGAANA--------------TDLYPRHFSRIMRKVV--
A0A0C9WMV0    TKS-KTCKLRQTKSASSSVSSSTGAGYA--------------TDLYPRHFSRIMRKVV--
                    *  :.                                   *.*.** *  .
```

ID		% Identity	Function	Organism
A0A137QF73	FGKSH----	43.9%	LPMO9	Leucoagaricus sp.
A0A0D2M3B5	--ESMY---	27.5%	LPMO9	Hypholoma sublateritium
A0A0C2XEH9	FGESIH---	40.5%	LPMO9	Hebeloma cylindrosporum
A0A067SZR5	FNGLSH---	42.5%	LPMO9	Galerina marginata
A0A0L6WMG1	HALSL----	34.9%	LPMO9	Termitomyces sp.
W4K048	HHP------	33.3%	LPMO9	Heterobasidion irregulare
I7DL47	HHS------	35.9%	LPMO9	Heterobasidion parviporum
R7SQR2	HHSS-----	41.0%	LPMO9	Dichomitus squalens
A0A067P992	FDRRRSW--	44.2%	LPMO9	Jaapia argillacea
**GtLPMO9D**	FQTTVHQW-	100.0%	LPMO9	Gloeophyllum trabeum
A0A0C3C9X4	FGSSF----	52.9%	LPMO9	Piloderma croceum
A8NLH8	ETGSFERRH	45.2%	LPMO9	Coprinopsis cinerea
A0A0C9WJR9	LGRSI----	36.6%	LPMO9	Laccaria amethystine
B0DGU6	LGRSI----	36.6%	LPMO9	Laccaria bicolor
B0CQE5	ARRSI----	29.3%	LPMO9	Laccaria bicolor
A0A0C9WMV0	ARRSI----	29.3%	LPMO9	Laccaria amethystine

Figure S4. Multiple sequence alignment of the C-terminus of *Gt*LPMO9D with similar C-termini of LPMOs. This domain contains two conserved motifs that are rich in positively charged amino acids. The second motif, with ca. every fourth amino acid being arginine/lysine, is likely to be an α-helix with a positive patch on one side. Aromatic amino acids (F, W, Y) are colored in green; positively charged amino acids (R, K) are colored in blue; negatively charged amino acids (D, E) are colored in red. The consensus symbols are indicated for the full alignment.

Section 3. Purification of GtLPMO9A-2 (Fig. S5).

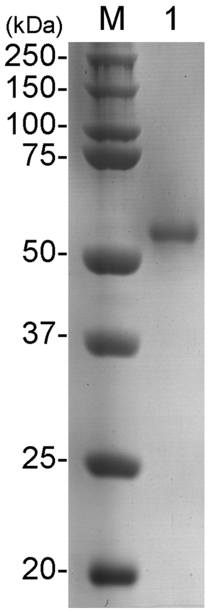

Figure S5. SDS-PAGE of recombinant *Gt*LPMO9A-2 produced in *Pichia pastoris*. Lane M, standard; Lane 1, purified recombinant *Gt*LPMO9A-2 treated with EndoH.

Section 4. Activity of GtLPMO9A-2 (Figs. S6-11).

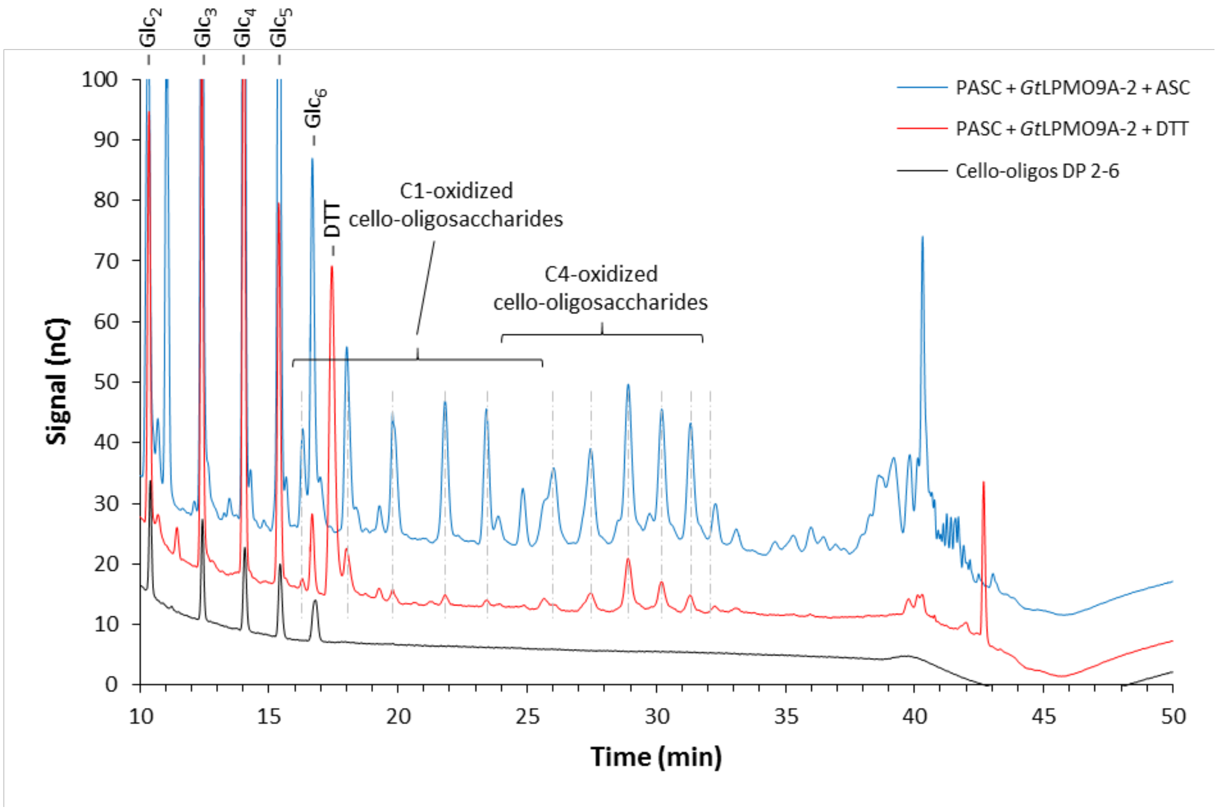

Figure S6. Comparison of the product profile of *Gt*LPMO9A-2 on PASC with ascorbic acid (ASC) or DTT as electron donor. The figure shows HPAEC-PAD chromatograms with cello-oligosaccharides released by *Gt*LPMO9A-2 from PASC; the peaks were assigned as in Fig. 3A. Grey dashed lines indicate oxidized oligosaccharides. The reaction conditions were as in Fig. 3.

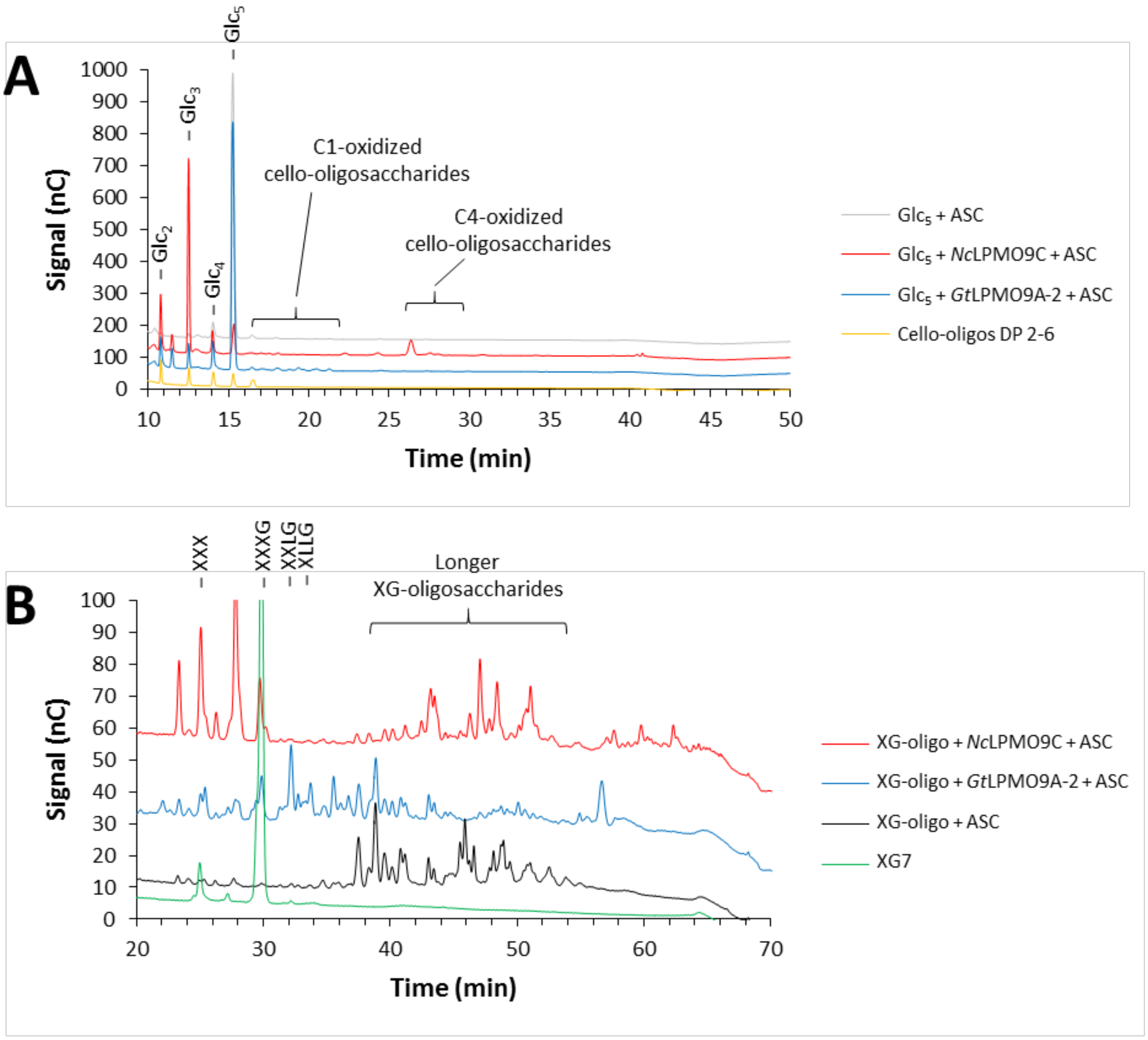

Figure S7. Products generated by *Gt*LPMO9A-2 or *Nc*LPMO9C from oligosaccharides. The figures show HPAEC-PAD chromatograms with peaks reflecting oligosaccharides released by *Gt*LPMO9A-2 (blue) or *Nc*LPMO9C (red) from cellopentaose **(A)** and xyloglucan oligosaccharides **(B)**. Peaks were assigned based on previous assignments by Isaksen et al. (2) and Agger et al. (3). Native cello-oligosaccharides are labeled as Glc_n where n is the degree of polymerization; xyloglucan oligosaccharides are labeled according to the nomenclature proposed by York et al. (4): X, glucose with xylose substitution; L, X with additional galactose substitution. Note that *Nc*LPMO9C is able to cleave cellopentaose (2), as is indeed visible in panel A.

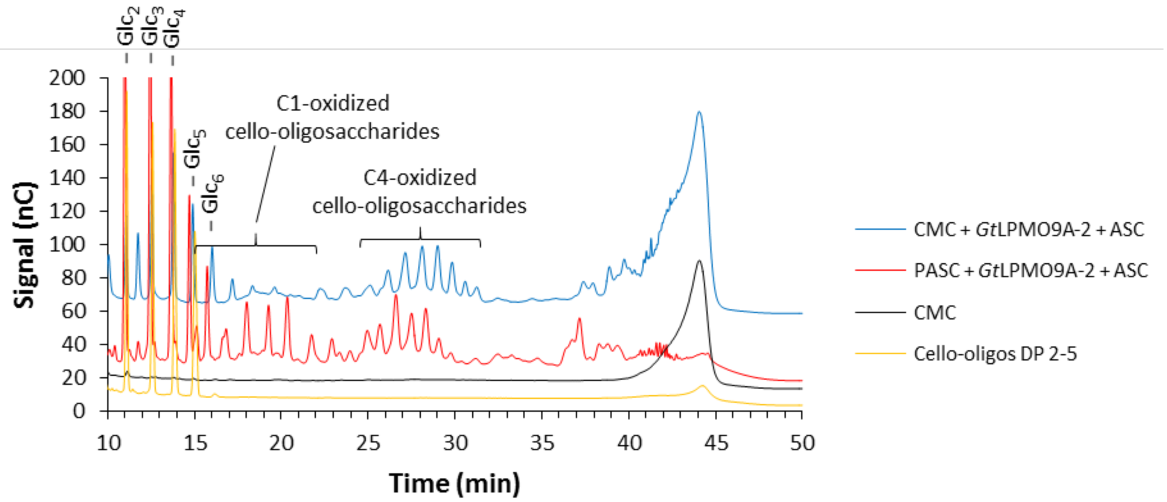

Figure S8. Products generated by *Gt*LPMO9A-2 or *Nc*LPMO9C from carboxymethylcellulose. HPAEC-PAD chromatograms showing oligosaccharides released by *Gt*LPMO9A-2 from CMC (blue) and PASC (red). Native cello-oligosaccharides with DP 2-5 (orange) were used as a standard.

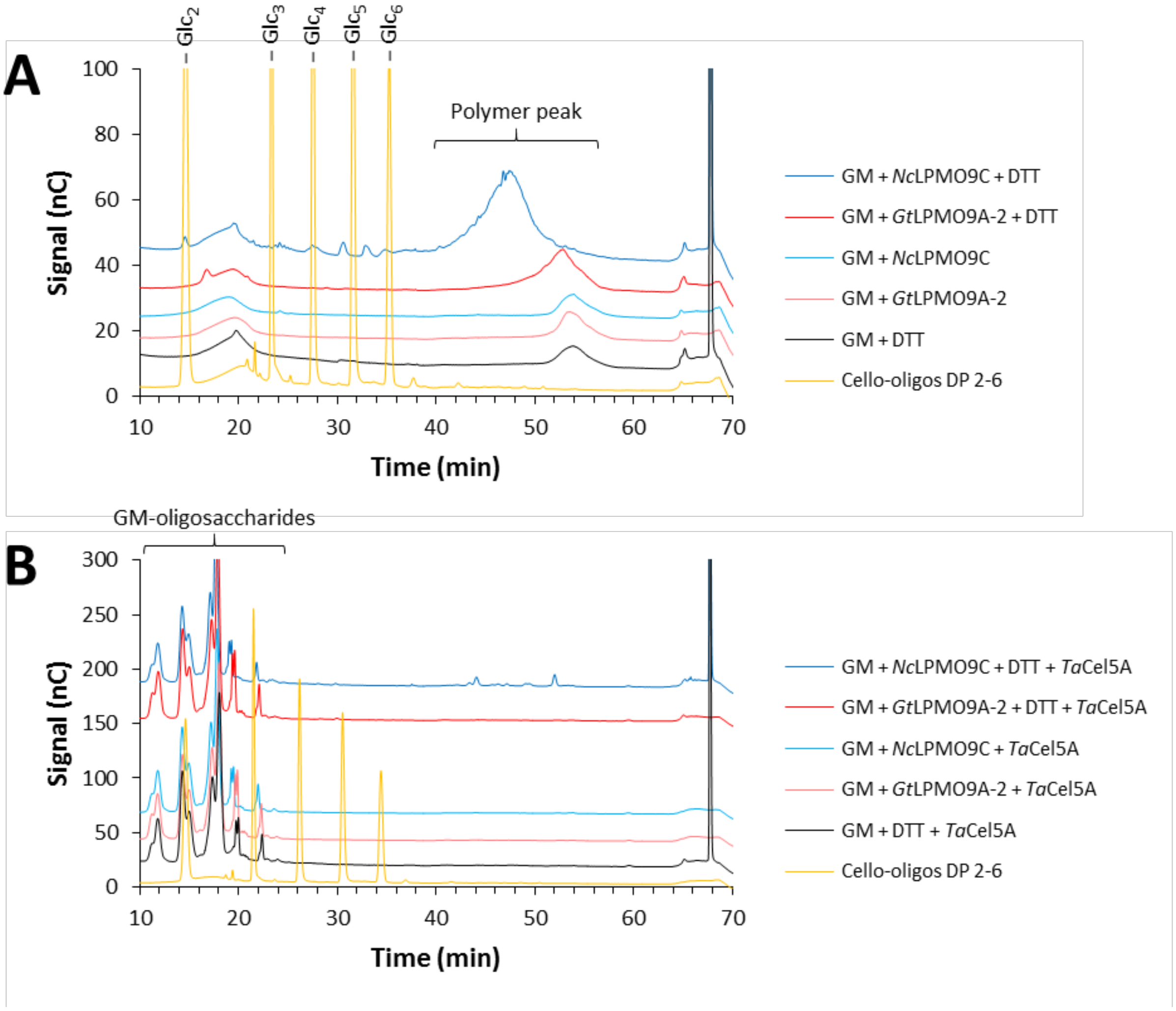

Figure S9. HPAEC-PAD analysis of reaction products generated from konjac glucomannan (GM) by *Gt*LPMO9A-2 or *Nc*LPMO9C in the dynamic viscosity experiments. Samples were taken after 16 hours of incubation and the chromatograms show the product profiles before **(A)** and after **(B)** a subsequent treatment with *Ta*Cel5A. Orange line: native cello-oligosaccharides with DP 2-6. Black line: GM incubated with DTT only. Red lines: products generated from GM by *Gt*LPMO9A-2 in the presence (dark red) or absence (light red) of DTT. Blue lines: products generated by *Nc*LPMO9C in the presence (dark blue) or absence (light blue) of DTT; the small peaks between 40 and 55 minutes visible in panel B for the reaction with *Nc*LPMO9C are likely

to be oxidized GM fragments. The reaction conditions are specified in the Materials and Methods section. Note that the chromatograms are identically scaled and that we thus observe an increase in the polymer detector signal upon partial depolymerization (panel A).

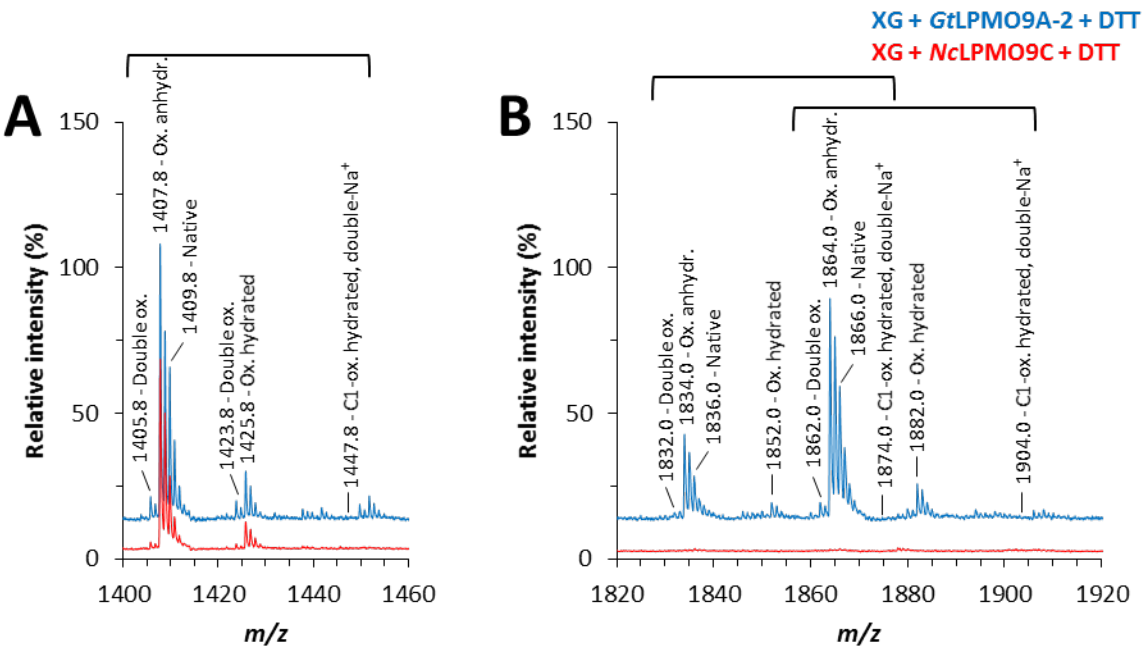

Figure S10. Details of the **(A)** Hex$_6$Pen$_3$ and **(B)** Hex$_7$Pen$_5$/Hex$_8$Pen$_4$ ion clusters in the MALDI-ToF spectrum for the end-point sample from the dynamic viscosity analysis where *Gt*LPMO9A-2 (blue lines) or *Nc*LPMO9C (red lines) reacted with tamarind xyloglucan (XG) in the presence of reducing agent (DTT). The clusters are marked with brackets; possible products in these clusters are the native (*m/z* 1409.8, 1836.0, and 1866.0), the C1-oxidized lactone or C4-oxidized ketoaldose (anhydrated species, *m/z* 1407.8, 1834.0, and 1864.0) and the C1-oxidized aldonic acid or C4-oxidized gemdiol (hydrated species, *m/z* 1425.8, 1852.0, and 1882.0). Signals corresponding to the double-oxidized species (*m/z* 1405.8, 1423.8, 1832.0, 1862.0) were very low, and signals corresponding to the sodium adduct of the aldonic acid sodium salt (*m/z* 1447.8, 1874.0, and 1904.0) could not be detected; the positions of these weak/absent signals are indicated.

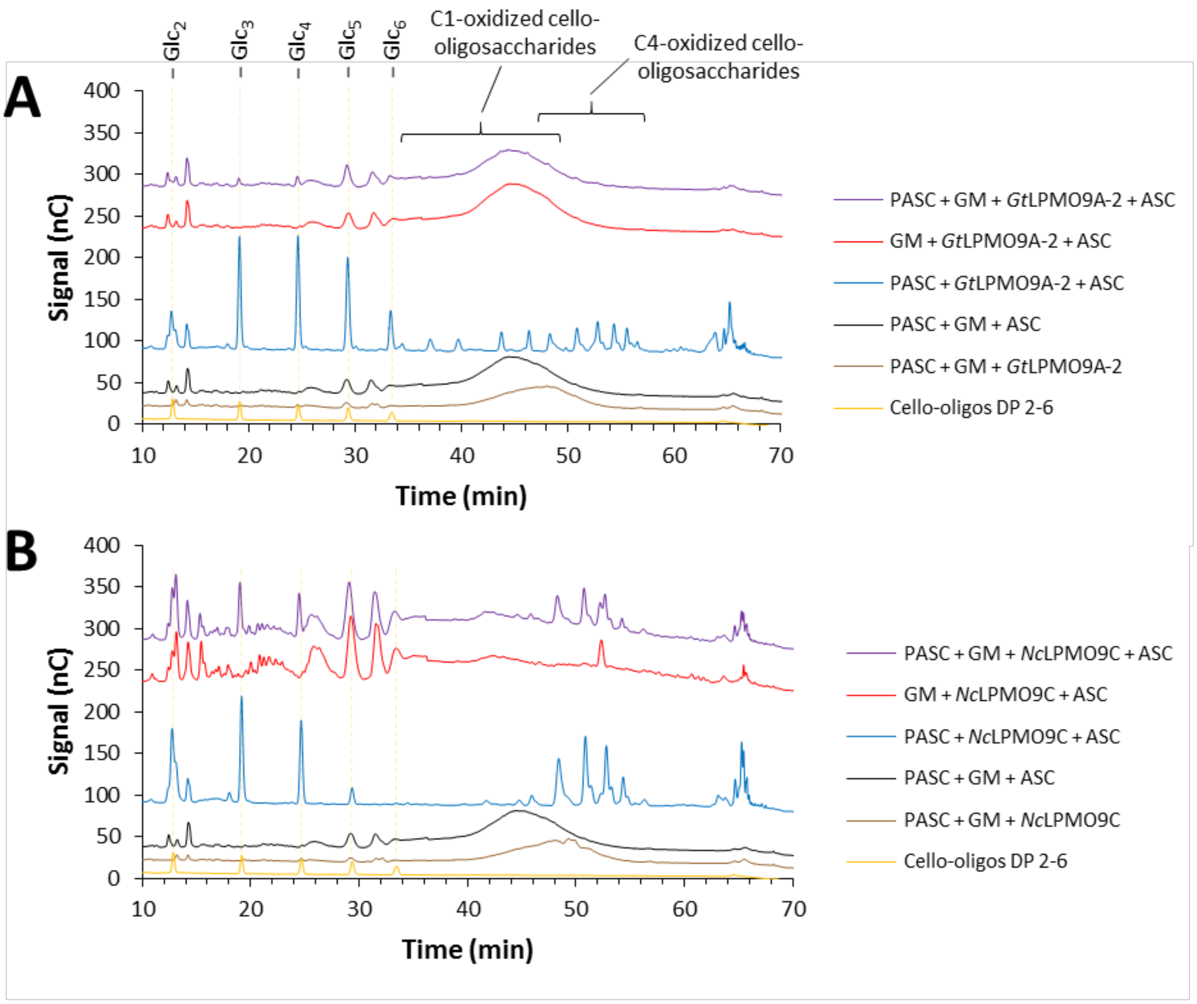

Figure S11. HPAEC-PAD analysis of reaction products generated by **(A)** *Gt*LPMO9A-2 or **(B)** *Nc*LPMO9C on glucomannan-coated PASC. Yellow line: native cello-oligosaccharides with DP 2-6. Brown line: mixture of PASC and konjac glucomannan (GM) incubated with LPMO only. Black line: mixture of PASC and GM incubated with ascorbic acid (ASC) only. Blue line: products generated from PASC with LPMO in the presence of ASC. Red line: products generated from GM with LPMO in the presence of ASC. Purple line: products generated from glucomannan-coated PASC with LPMO in the presence of ASC. The reaction conditions are specified in the Materials and Methods section.

References

1. **Jagadeeswaran G, Gainey L, Prade R, Mort AJ.** 2016. A family of AA9 lytic polysaccharide monooxygenases in *Aspergillus nidulans* is differentially regulated by multiple substrates and at least one is active on cellulose and xyloglucan. Appl Microbiol Biotechnol **100:**4535-4547.

2. **Isaksen T, Westereng B, Aachmann FL, Agger JW, Kracher D, Kittl R, Ludwig R, Haltrich D, Eijsink VG, Horn SJ.** 2014. A C4-oxidizing lytic polysaccharide monooxygenase cleaving both cellulose and cello-oligosaccharides. J Biol Chem **289:**2632-2642.

3. **Agger JW, Isaksen T, Várnai A, Vidal-Melgosa S, Willats WGT, Ludwig R, Horn SJ, Eijsink VGH, Westereng B.** 2014. Discovery of LPMO activity on hemicelluloses shows the importance of oxidative processes in plant cell wall degradation. Proc Natl Acad Sci U S A **111:**6287-6292.

4. **York WS, van Halbeek H, Darvill AG, Albersheim P.** 1990. Structural analysis of xyloglucan oligosaccharides by 1H-n.m.r. spectroscopy and fast-atom-bombardment mass spectrometry. Carbohydr Res **200:**9-31.
